# GNE‐317 Reverses MSN‐Mediated Proneural‐to‐Mesenchymal Transition and Suppresses Chemoradiotherapy Resistance in Glioblastoma via PI3K/mTOR

**DOI:** 10.1002/advs.202412517

**Published:** 2025-02-07

**Authors:** Yong‐Chang Yang, Xing‐Yu Jin, Ling‐Ling Yang, Xing Xu, Yang Xie, Yi‐Ding Ai, Xin‐Chao Li, Ye‐Cheng Ma, Cheng‐Long Xu, Qi Li, Xiang‐Lian Ge, Tai‐Long Yi, Tao Jiang, Xiao‐Guang Wang, Ying‐Zhe Piao, Xun Jin

**Affiliations:** ^1^ Department of Biochemistry and Molecular Biology Tianjin Medical University Cancer Institute and Hospital National Clinical Research Center for Cancer Key Laboratory of Cancer Prevention and Therapy Tianjin Tianjin's Clinical Research Center for Cancer Huanhuxi Road, Ti‐Yuan‐Bei Hexi District Tianjin 300060 P. R. China; ^2^ Tianjin Medical University Tianjin 300060 P. R. China; ^3^ Beijing Neurosurgical Institute Capital Medical University Beijing 100054 P. R. China; ^4^ Department of Neuro‐Oncology and Neurosurgery Tianjin Medical University Cancer Institute and Hospital National Clinical Research Center for Cancer Key Laboratory of Cancer Prevention and Therapy Tianjin 300060 P. R. China

**Keywords:** chemoradiotherapy resistance, glioblastoma, GNE‐317, MSN, PI3K/mTOR, proneural‐to‐mesenchymal transition

## Abstract

Glioblastoma (GBM) resistance to chemoradiotherapy is a major factor contributing to poor treatment outcomes. This resistance markedly affects the effectiveness of surgery combined with chemoradiotherapy and leads to post‐surgical tumor recurrence. Therefore, exploring the mechanisms underlying chemoradiotherapy resistance in GBM is crucial for understanding its progression and improving therapeutic options. This study found that moesin (MSN) acts as a key promotor of chemoradiotherapy resistance in glioma stem cells (GSCs), enhancing their proliferation and stemness maintenance. Mechanistically, MSN activates the downstream PI3K/mTOR signaling pathway, driving the proneural‐to‐mesenchymal transition (PMT) in GSCs. This process enhances the repair of DNA damage caused by radiotherapy (RT) and temozolomide (TMZ), thereby increasing the resistance of GSCs to chemoradiotherapy. Additionally, GNE‐317, a small molecule drug capable of crossing the blood‐brain barrier, specifically inhibits MSN and suppresses the activation of downstream PI3K/mTOR signaling. Importantly, the combination of GNE‐317 with RT and TMZ exhibits a strong synergistic effect both in vivo and in vitro, achieving better efficacy compared to the traditional combination of RT and TMZ. This study not only advances understanding of the mechanisms underlying chemoradiotherapy resistance in GBM but also provides a promising new approach for enhancing treatment outcomes.

## Graphical abstract

The study identifies MSN as a key regulator of proneural‐to‐mesenchymal transition (PMT) in GSCs, driving resistance to radiotherapy (RT) and temozolomide (TMZ) in glioblastoma (GBM). However, GNE‐317 specifically inhibits MSN, reverses PMT, resensitizes cells to therapy, and shows promise as a targeted strategy to overcome chemoradiotherapy resistance in GBM.

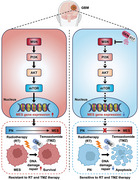

## 1 Introduction

Glioblastoma (GBM) represents the predominant and most virulent primary malignancy affecting the central nervous system (CNS), ranking as a principal factor in patient deaths.^[^ ^1^, ^2^ ^]^ Despite maximal surgical resection followed by chemoradiotherapy for patients with newly diagnosed GBM, tumor recurrence is nearly inevitable and treatment options are limited.^[^ ^3^, ^4^ ^]^ The median overall survival for these individuals ranges below 15 months, while for recurrent glioblastoma, it is ≈2 to 9 months.^[^ ^5^ ^]^ The challenges in GBM treatment arise not only from the limitations of safe surgical resection margins but also from resistance to adjuvant chemoradiotherapy, including radiotherapy (RT) combined with temozolomide (TMZ).^[^ ^6^ ^]^ Therefore, understanding the mechanisms underlying chemoradiotherapy resistance is critical for improving current treatments and developing more effective therapeutic strategies.

Chemoradiotherapy resistance in GBM is driven by several factors, with tumor heterogeneity and cellular plasticity playing key roles in treatment resistance and recurrence.^[^ ^7^ ^]^ Large‐scale genomic analyses of histologically similar GBM samples have identified three major molecular subtypes: proneural (PN), classical (CL), and mesenchymal (MES).^[^ ^8^ ^]^ Notably, the PN and MES subtypes differ markedly in prevalence, prognosis, and treatment response; the PN subtype is often linked to favorable outcomes and treatment sensitivity while the MES subtype is associated with poor prognosis and greater resistance to therapy.^[^ ^9^ ^]^ Additionally, GBMs contain populations of glioma stem cells (GSCs), which exhibit self‐renewal and multipotent differentiation capabilities^[^ ^10^ ^]^ and are key drivers of tumor proliferation, recurrence, and resistance to chemoradiotherapy.^[^ ^11^ ^]^ Furthermore, GSCs expressing markers of the PN and MES transcriptomic subtypes can be derived from GBMs.^[^ ^12^ ^]^ However, GSCs expressing markers of the CL transcriptomic subtypes have not been convincingly identified.^[^ ^13^ ^]^ Similarly, GSCs derived from these GBM subtypes display corresponding characteristics, with MES GSCs being more resistant to treatment.^[^ ^14^, ^15^, ^16^ ^]^ Therefore, GSCs are an ideal model for studying GBM therapies. Current research indicates that as GBM progresses and recurs, GSCs undergo proneural‐to‐mesenchymal transition (PMT), enhancing their resistance to therapeutic stressors.^[^ ^17^, ^18^, ^19^ ^]^ As a result, the PMT process is considered to be a significant driver of chemoradiotherapy resistance and tumor recurrence.

Several potential mechanisms have been implicated in the initiation of PMT. For example, one groundbreaking study identified CEBPB and STAT3 as key synergistic regulators of mesenchymal transformation.^[^ ^20^ ^]^ The PMT process also encompasses stimulation of both NF‐KB and TAZ signaling cascades.^[^ ^9^, ^21^ ^]^ Furthermore, interactions between tumor‐associated macrophages and GBM cells within the tumor microenvironment can promote PMT.^[^ ^22^ ^]^ However, despite extensive research, the molecular mechanisms underlying PMT in GSCs remain poorly understood. Targeting the key factors of the PMT process to enhance the efficacy of RT and TMZ represents a promising strategy for overcoming chemoradiotherapy resistance in GBM.

In this study, we identified MSN as a key gene upregulated in patients with chemoradiotherapy‐resistant GBM. Moreover, we demonstrated that MSN not only promoted GSC proliferation and stemness but also increased resistance to RT and TMZ. Furthermore, MSN was closely associated with the MES subtype, and silencing MSN in GSCs reversed PMT by inhibiting the PI3K/mTOR pathway. Importantly, through a small‐molecule drug screen, we identified GNE‐317 as a PI3K/mTOR inhibitor capable of crossing the blood‐brain barrier (BBB), which not only effectively suppressed MSN expression but also exhibited a strong synergistic effect with RT and TMZ, significantly enhancing the efficacy of these combined therapies.^[^ ^23^ ^]^

## 2 Results

### 2.1 MSN is Identified as a Key Factor Causing Resistance to Chemoradiotherapy in GBM

**Figure 1:**
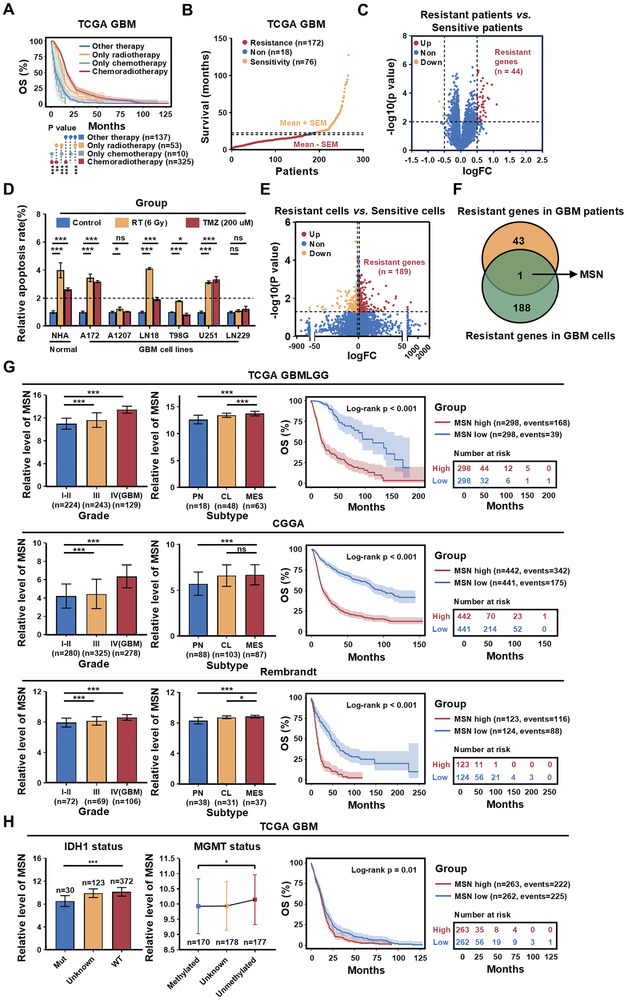
MSN may be a key factor in chemoradiotherapy resistance in GBM. A) Kaplan‐Meier survival curves showing the association between standard chemoradiotherapy and prognosis in GBM patients in the TCGA GBM database. (Other therapy, *n* = 137; Only radiotherapy, *n* = 53; Only chemotherapy, *n* = 10; Chemoradiotherapy, *n* = 325). *P* values were calculated using the log‐rank test. ^**^ *p* < 0.01, ^***^ *p* < 0.001. B) Scatter plot of patients who received standard chemoradiotherapy and had an outcome of death (*n* = 266), divided into sensitivity (*n* = 76) and resistance (*n* = 172) groups based on survival time. Data are shown as mean ± SEM. C) Volcano plot comparing resistant and sensitive patients. Red indicates chemoradiotherapy‐resistant genes, and yellow indicates chemoradiotherapy‐sensitive genes. The x‐axis represents log2(fold change), and the y‐axis represents ‐log10(*p* value). D) Quantification of apoptosis levels in one normal cell line and six GBM cell lines after control, RT, or TMZ treatment. Data are shown as mean ± SD. ^*^ *p* < 0.05, ^***^ *p* < 0.001, ns, *p* > 0.05; two‐tailed unpaired *t*‐test. E) Volcano plot showing differential genes between resistant and sensitive cells. Red indicates chemoradiotherapy‐resistant genes, and yellow indicates chemoradiotherapy‐sensitive genes. F) Venn diagram showing chemoradiotherapy‐resistant genes common to both GBM cells and GBM patients. G) MSN expression in different grades and subtypes, and its association with survival outcomes in the glioma database (TCGA GBMLGG, CGGA, and Rembrandt). Bar chart data are shown as mean ± SD. ^*^ *p* < 0.05, ^***^ *p* < 0.001, ns, *p* > 0.05; two‐tailed unpaired *t*‐test. For survival analysis, *p* values were calculated using the log‐rank test. H) MSN expression and prognosis in GBM patients with different IDH1 and MGMT status. Bar and box chart data are shown as mean ± SD. ^*^ *p* < 0.05, ^***^ *p* < 0.001; two‐tailed unpaired *t*‐test. For survival analysis, *p* values were calculated using the log‐rank test.

Standard chemoradiotherapy offers limited improvement in GBM patient prognosis, with its overall effectiveness remaining suboptimal (**Figure** **1**A). To quantify the impact of chemoradiotherapy on GBM, we used data from patients in The Cancer Genome Atlas (TCGA) GBM database who had received standard chemoradiotherapy and had an outcome of death. These patients were divided into chemoradiotherapy‐resistant and chemoradiotherapy‐sensitive groups based on their survival time (Figure 1B). Differential analysis between the resistant and sensitive groups revealed a set of genes associated with chemoradiotherapy resistance (Figure 1C). Next, we treated GBM cells in vitro with RT and TMZ for 48 h and classified them into chemoradiotherapy‐resistant and ‐sensitive cells based on their apoptosis rates (Figure 1D; Figure S1, Supporting Information). Differential analysis of the two groups identified a gene signature (*n* = 189) linked to chemoradiotherapy resistance in GBM cells (Figure 1E). By overlapping the two gene sets, we identified MSN as a gene associated with chemoradiotherapy resistance in patients with GBM and in GBM cells (Figure 1F). Additionally, in public databases, MSN also played a significant role in gliomas, particularly in high‐grade gliomas such as GBM (Figure 1G,H).

**Figure 2:**
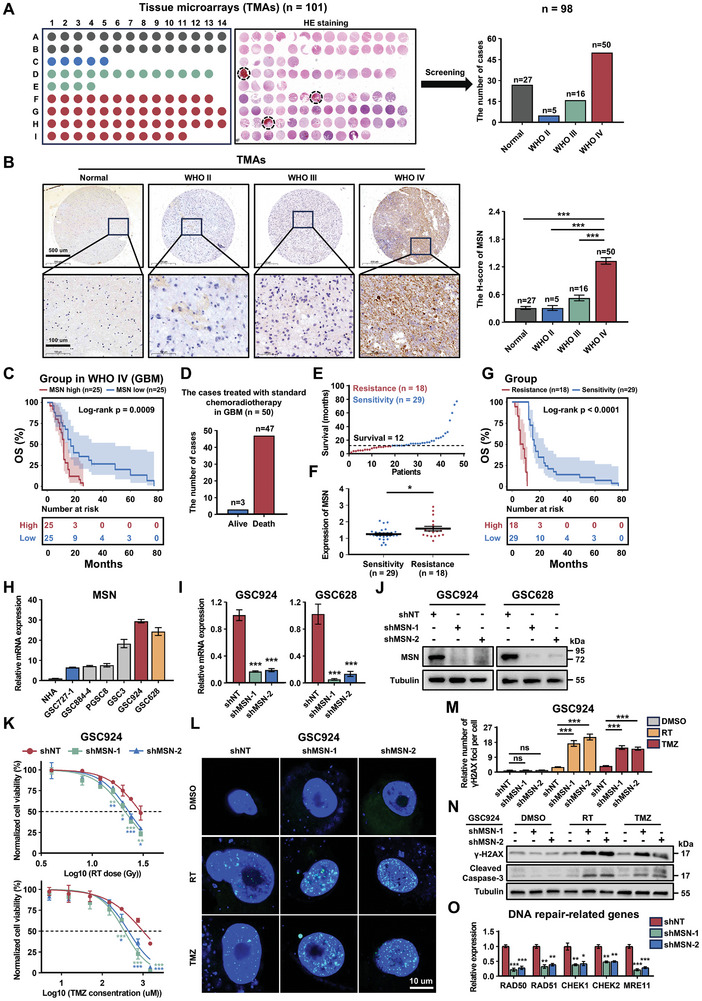
MSN enhances chemoradiotherapy resistance in GBM patients and GSCs. A) Grade information and HE staining of glioma TMAs. Black circles indicate TMA spots excluded due to hemorrhage. B) Representative images and quantification of MSN expression in TMAs, grouped by glioma grades. Data are shown as mean ± SEM. ^***^ *p* < 0.001; two‐tailed unpaired *t*‐test. C) Kaplan‐Meier survival curves for patients stratified by median MSN expression levels in grade IV glioma (GBM). Censored data are represented by vertical lines. *P* values were calculated using the log‐rank test. D) Bar chart showing the survival status of GBM patients after standard chemoradiotherapy. E) Scatter plot showing patients who received standard chemoradiotherapy and had an outcome of death, divided into chemoradiotherapy‐sensitive and chemoradiotherapy‐resistant groups based on median survival time. F) Scatter plot showing MSN expression levels in chemoradiotherapy‐sensitive and chemoradiotherapy‐resistant groups. Data are shown as mean ± SEM. ^*^ *p* < 0.05; two‐tailed unpaired *t*‐test. G) Kaplan‐Meier survival curves for chemoradiotherapy‐sensitive and chemoradiotherapy‐resistant groups. *P* values were calculated using the log‐rank test. H) Bar graph showing relative mRNA expression levels of MSN in normal tissues and GSCs by qPCR. Data are shown as mean ± SEM. *n* = 3 independent experiments. I) qPCR and J) immunoblot analysis of MSN expression in GSCs transduced with non‐targeting shRNA (shNT) or MSN shRNA (shMSN). qPCR data are shown as mean ± SEM. *n* = 3 independent experiments. ^***^ *p* < 0.001; two‐tailed unpaired *t*‐test. K) Cell viability assay of GSC924 transduced with shNT or shMSN, treated with different doses of RT or varying concentrations of TMZ for 48 h. *n* = 3 biological independent samples. The black dashed line represents the IC50 value. ^*^ *p* < 0.02, ^**^ *p* < 0.01, ^***^ *p* < 0.001; two‐way ANOVA followed by Tukey's multiple comparison test. L) Representative images and M) quantification of γ‐H2AX staining in GSC924 with or without RT treatment (6 Gy, 48 h) or TMZ (200 µm, 48 h). Scale bar: 10 µm. Data are shown as mean ± SEM. *n* = 5 independent experiments. ^***^ *p* < 0.001, ns, *p* > 0.05; two‐tailed unpaired *t*‐test. N) Immunoblot analysis of γ‐H2AX and cleaved Caspase‐3 expression in GSC924 with or without RT (6 Gy, 48 h) or TMZ (200 µm, 48 h) treatment. O) Relative mRNA levels of DNA repair‐related genes in GSC924 with or without RT (6 Gy, 48 h) or TMZ (200 µm, 48 h) treatment. Data are shown as mean ± SEM. *n* = 3 independent experiments. ^*^ *p* < 0.05, ^**^ *p* < 0.01, ^***^ *p* < 0.001; two‐tailed unpaired *t*‐test.

To further validate our findings, MSN expression was assessed using glioma tissue microarrays (TMAs) (**Figure** **2**A). The findings demonstrated that MSN exhibited elevated expression in GBM tissues and correlated with unfavorable outcomes (Figure 2B,C). We then categorized patients with GBM who had received standard chemoradiotherapy and had an outcome of death into chemoradiotherapy‐resistant and ‐sensitive groups based on the median survival time (Figure 2D,E). We found that MSN was highly expressed in the chemoradiotherapy‐resistant group and that these patients had a poorer prognosis than those in the chemoradiotherapy‐sensitive group (Figure 2F,G). These results suggest that MSN promotes chemoradiotherapy resistance in patients with GBM. We also investigated the role of MSN using GSC models. First, we selected GSC924 and GSC628 cells, which have high MSN expression, as positive cells for our experiments (Figure 2H). Subsequently, MSN was knocked down in GSC924 and GSC628 cells (Figure 2I,J). After MSN knockdown, half maximal inhibitory concentration (IC50) values for RT and TMZ treatment were significantly reduced, further inhibiting GSC growth (Figure 2K; Figure S2A, Supporting Information). Furthermore, in the MSN knockdown group, RT and TMZ treatment significantly reduced the DNA damage repair capacity of GSCs, resulting in notable apoptosis (Figure 2L–O; Figure S2B–E, Supporting Information). All experimental results suggest that MSN enhances chemoradiotherapy resistance in both GBMs and GSCs.

### 2.2 MSN Supports the Growth and Maintenance of Stemness in Glioma Stem Cells

**Figure 3:**
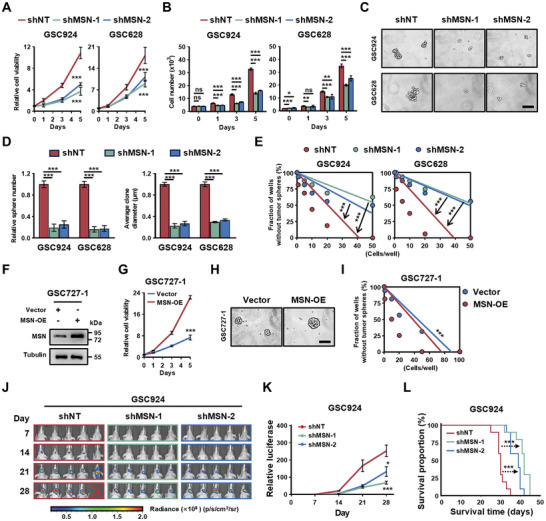
MSN is required for GSC proliferation and self‐renewal. A) Cell viability assay of GSCs transduced with shNT or shMSN. *n* = 6 (GSC924) or *n* = 6 (GSC628) biological independent samples. Data are shown as mean ± SD. ^***^ *p* < 0.001, two‐way ANOVA followed by Tukey's multiple comparison test. B) Two independent shRNAs targeting MSN reduced the growth of GSC924 and GSC628 compared to shNT, as measured by cell count. Data are shown as mean ± SD from 5 independent experiments. ^*^ *p* < 0.05, ^**^ *p* < 0.01, ^***^ *p* < 0.001, ns, *p* > 0.05; two‐way ANOVA followed by Tukey's multiple comparison test. C) Representative images of tumorspheres from GSCs transduced with shNT or shMSN. Scale bar: 150 µm. D) Quantification of tumorsphere numbers and average clone diameter in GSCs transduced with shNT or shMSN. *n* = 16 for tumorsphere number, *n* = 5 biological independent cell cultures for average clone diameter. Data are shown as mean ± SEM. ^***^ *p* < 0.001; two‐tailed unpaired *t*‐test. E) In vitro extreme limiting dilution analysis of tumorsphere formation in GSCs expressing shNT or shMSN. *n* = 16 biological independent cell cultures. ^***^ *p* < 0.001 by ELDA analysis. F) Immunoblot analysis of MSN expression in GSC727‐1‐Vector and GSC727‐1‐MSN‐overexpression (MSN‐OE) through lentiviral infection. G) Cell viability assay, H) representative images of tumorspheres, and I) extreme limiting dilution analysis of tumorsphere formation in GSC727‐1 expressing vector or MSN‐OE. J) Representative images and K) quantification of relative luciferase on days 7, 14, 21, and 28 post‐transplantation; bioluminescence is measured in p^−1^s^−1^cm^2‐1^sr. GSC924: shNT (*n* = 5), shMSN‐1 (*n* = 5), shMSN‐2 (*n* = 5). Data are shown as mean ± SEM. ^*^ *p* < 0.05, ^***^ *p* < 0.001; one‐way ANOVA followed by Tukey's multiple comparison test. L) Kaplan‐Meier survival curves of mice bearing GSC924‐derived xenografts expressing shNT or shMSN. ^***^ *p* < 0.001, log‐rank test. GSC924: shNT (*n* = 10), shMSN‐1 (*n* = 10), shMSN‐2 (*n* = 10).

Given MSN's role in chemoradiotherapy resistance, we explored its effects on GSC‐driven tumor progression both in vitro and in vivo. In vitro, silencing MSN in GSC924 and GSC628 inhibited proliferation, reduced cell viability, and impaired self‐renewal capacity, as demonstrated by diminished neurosphere formation and results from limiting dilution assays (**Figure** **3**A–E). In contrast, MSN overexpression enhanced both the growth and stemness of GSC727‐1, which are typically negative cells with low MSN expression (Figure 3F–I). Given MSN's essential function in GSCs, potentially facilitating aggressive expansion, we investigated the consequences of MSN disruption on GSC‐driven tumor growth in vivo. GSCs (GSC924 and GSC628) expressing firefly luciferase, combined with either MSN shRNA or control shRNA (shNT), were transplanted into the brains of immunocompromised mice. Bioluminescence imaging revealed that MSN knockdown significantly inhibited GSC‐driven tumor growth (Figure 3J–K; Figures S3A,B, Supporting Information). Consequently, mice with xenografts from GSCs expressing shMSN exhibited significantly longer survival than did control mice (Figure 3L, Figure S3C, Supporting Information). Together, these findings highlight that MSN is indispensable for GSC proliferation and maintenance of stemness, both in vitro and in vivo, driving tumor growth in GBM.

### 2.3 High‐MSN Chemoradiotherapy Resistant GBM Subclusters are Localized to MES‐Like Regions

**Figure 4:**
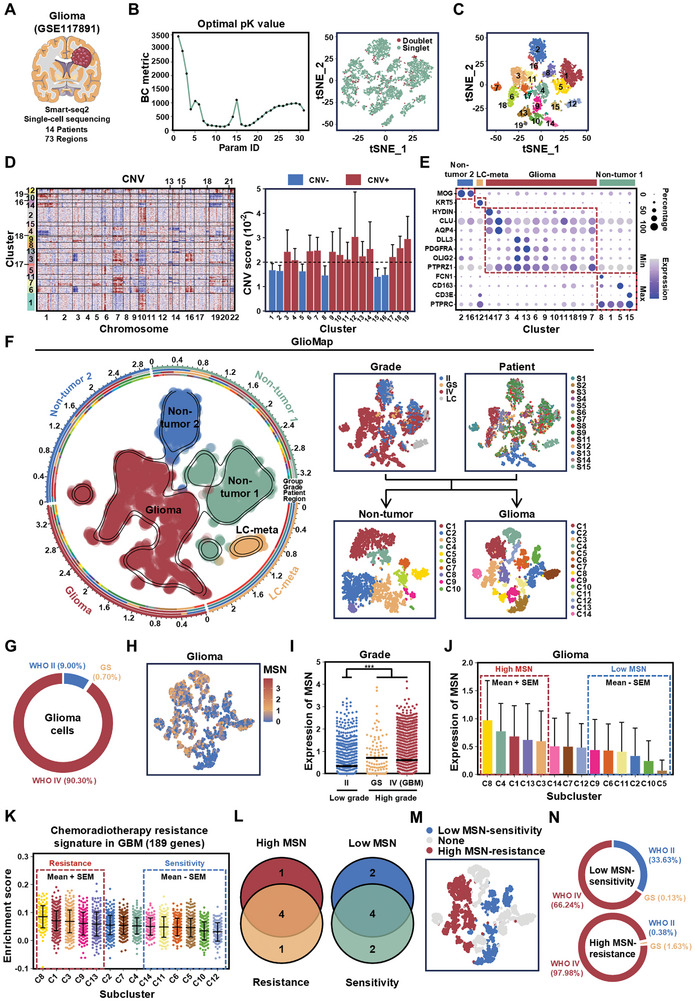
At the single‐cell level, MSN is highly correlated with chemoradiotherapy resistance. A) Basic information about single‐cell sequencing. B) Cell filtering excludes non‐single cells using DoubletFinder. C) t‐SNE analysis of all cells, showing 19 significant cell clusters color‐coded and labeled accordingly. D) RNA‐derived single‐cell CNV information. Cell clusters were classified as CNV^−^ and CNV^+^ based on CNV score. E) Dot plot of cell clusters, showing shared and specific marker genes, dividing clusters into non‐tumor 1, non‐tumor 2, glioma, and lung cancer metastasis (LC‐meta) cells. F) t‐SNE visualization of the Gliomap, including patient data, grades (WHO II, Gliosarcoma (GS), and WHO IV), and subdivisions of non‐tumor and glioma subclusters. The axis outside the circular plot displays the log scale of total cell numbers for each cell type (level‐3 annotation). G) Pie chart showing the proportion of glioma cells originating from patients of different grades. H) Distribution of MSN expression on the original t‐SNE coordinates for 14 glioma subclusters. I) Scatter plot showing MSN expression levels in patients with low (WHO II) and high grades (GS and WHO IV). The black line represents the mean value. ^***^ *p* < 0.001, two‐tailed unpaired *t*‐test. J) Bar chart showing MSN expression across 14 glioma subclusters, categorized as high MSN (mean + SEM) and low MSN (mean – SEM) groups. K) Scatter plot showing enrichment scores of the chemoradiotherapy‐resistance signature, categorizing glioma subclusters into chemoradiotherapy‐resistant (mean + SEM) and chemoradiotherapy‐sensitive (mean – SEM) groups. L) Venn diagram showing high‐MSN chemoradiotherapy‐resistant and low‐MSN chemoradiotherapy‐sensitive glioma subclusters. M) Distribution of high‐MSN‐resistance and low‐MSN‐sensitivity groups on the original t‐SNE coordinates. N) Pie charts showing the proportions of cells from low‐MSN chemoradiotherapy‐sensitive and high‐MSN chemoradiotherapy‐resistant glioma subclusters in glioma patients of different grades.

Given the heterogeneity of GBM and the varying degrees of chemoradiotherapy resistance, we employed single‐cell sequencing to characterize resistant GBM cell clusters. To more precisely identify the gene expression patterns of different tumor subclusters, we need a tool that can provide full‐length transcript information and detect low‐abundance gene expression at single‐cell resolution. Therefore, we used Smart‐seq2 single‐cell sequencing data from 73 regions across 14 patients (**Figure** **4**A; Figure S4A,B, Supporting Information). Following data quality control and the use of predicted copy number variation (CNV) and marker gene expression, we constructed a Gliomap with information on four factors: grade, patient, non‐tumor, and glioma (Figure 4B–F; Figure S5A–D, Supporting Information). Among glioma cells, 90.3% were from WHO grade IV patients, 0.7% were from gliosarcoma (GS), and 9% were from WHO grade II patients (Figure 4G). We found that MSN was highly expressed in high‐grade glioma cells (both GBM and GS) (Figure 4H–I). Therefore, we divided the glioma clusters into high and low groups based on MSN expression levels (Figure 4J). Next, using the GBM chemoradiotherapy resistance signature (*n* = 189) defined in Figure 1E, we performed gene set enrichment analysis across 13 glioma clusters. Based on the results of this enrichment, we classified the glioma cells into chemoradiotherapy‐resistant and chemoradiotherapy‐sensitive groups (Figure 4K). As expected, the high MSN group showed a significant overlap with the chemoradiotherapy‐resistant group (Figure 4L). Thus, we were able to identify high MSN/chemoradiotherapy‐resistant and low MSN/chemoradiotherapy‐sensitive glioma subclusters (Figure 4M). Moreover, compared to the low MSN‐sensitive subclusters, the cells in the high MSN‐resistant subclusters were almost exclusively derived from GBM (Figure 4N). Intriguingly, the high MSN‐resistant subclusters are characterized by enhanced survival signaling, metabolic reprogramming (including glycolysis and fatty acid metabolism), and resistance to apoptosis. These features are driven by key pathways such as PI3K/AKT/mTOR, hypoxia/HIF‐1, NF‐kB, and TGF‐β/Smad, collectively highlighting their pivotal role in GBM chemoradiotherapy resistance (Figure S6A–D, Supporting Information).

**Figure 5:**
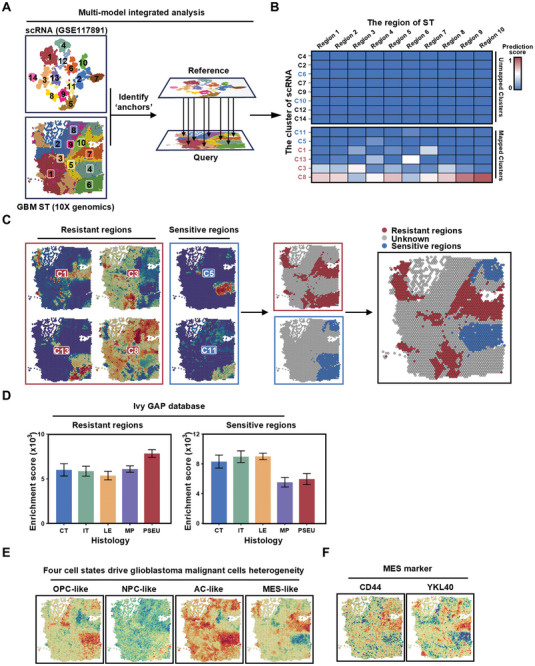
Combined analysis indicates that high‐MSN chemoradiotherapy‐resistant cell subclusters are predominantly distributed in the MES‐like cells region. A) Schematic of integrated analysis combining glioma single‐cell sequencing and GBM spatial transcriptomics. B) The heatmap shows the matching between scRNA clusters and spatial transcriptomics data. In the scRNA clusters, red clusters indicate high‐MSN chemoradiotherapy‐resistant groups, while blue clusters represent low‐MSN chemoradiotherapy‐sensitive groups. C) Spatial distribution of high‐MSN chemoradiotherapy‐resistant and low‐MSN chemoradiotherapy‐sensitive subclusters. D) The bar chart represents the enrichment scores of high‐MSN chemoradiotherapy‐resistant and low‐MSN chemoradiotherapy‐sensitive groups in different regions of the Ivy GAP database. CT: Cellular Tµmor; IT: Infiltrating Tµmor; LE: Leading Edge; MP: Microvascular Proliferation; PSEU: Pseudopalisading cells. Data are presented as mean ± SD. Spatial distribution of E) 4 cell states scores defined by scRNA‐seq and F) MES marker.

The spatial distribution of cells within tumor tissue also determines the functions they exert. Therefore, we integrated single‐cell sequencing with spatial transcriptomics of GBM and mapped the high MSN/resistant GBM subclusters to spatial locations within the tumor (**Figure** **5**A,B). Using this approach, we identified regions potentially associated with chemoradiotherapy resistance (Figure 5C). To explore the anatomical locations of these regions, we performed a joint analysis using the Ivy Glioblastoma Atlas Project (Ivy GAP) database.^[^ ^24^ ^]^ The results showed that, compared to the sensitive regions, the resistant regions were primarily located in the microvascular proliferation (MP) and pseudopalisading cells (PSEU) regions (Figure 5D). These regions not only exhibit intense hypoxia, increased angiogenesis, and abnormal blood vessel networks, but according to previous studies, they are also regions where MES GSCs are frequently enriched.^[^ ^25^, ^26^, ^27^ ^]^ This observation was further validated using our spatial transcriptomic data (Figure 5E,F). The above results indicate a significant association between MSN and the GBM MES subtype, suggesting that MSN may promote chemoradiotherapy resistance by maintaining the characteristics of the MES subtype.

### 2.4 MSN Triggers the PMT, Enhancing GBM Resistance to Chemoradiotherapy

**Figure 6:**
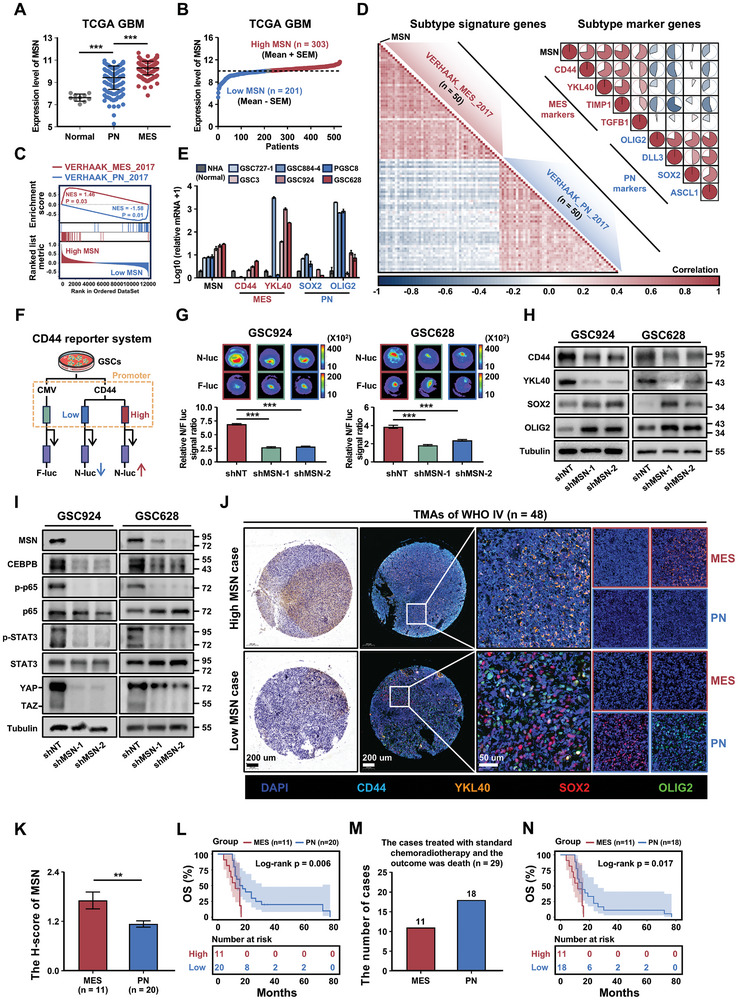
MSN promotes the proneural‐to‐mesenchymal transition to resist chemoradiotherapy. A) The scatter plot shows MSN expression levels across different GBM subtypes in the TCGA GBM database. Data are presented as mean ± SD. ^***^ *p* < 0.001, two‐tailed unpaired *t*‐test. B) The scatter plot divides patients into high‐MSN (Mean + SEM) and low‐MSN (Mean – SEM) groups based on MSN expression levels. C) GSEA enrichment analysis of high‐MSN and low‐MSN groups in the TCGA GBM database. D) The left heatmap shows the correlation analysis between MSN and subtype signature genes, while the right pie chart represents the correlation analysis between MSN and subtype marker genes. E) qPCR analysis is used to determine the subtypes of different GSCs. F) Schematic of the CD44 reporter system. G) Representative images and quantitative analysis of CD44 reporter system luciferase in GSCs transduced with shNT or shMSN. *n* = 5 (GSC924) or *n* = 5 (GSC628) biological independent samples. Data are presented as mean ± SEM. ^***^ *p* < 0.001, two‐tailed unpaired *t*‐test. H) Immunoblot analysis of subtype markers in GSCs transduced with shNT or shMSN. I) Immunoblot analysis of key MES transcriptional regulators and pathways in GSCs transduced with shNT or shMSN. J) Multiplex immunofluorescence staining of TMAs in GBM, dividing GBM patients into MES or PN subtypes. The left side shows the immunohistochemical staining of MSN. K) The bar chart shows the MSN H‐score in MES and PN subtype patients. Data are presented as mean ± SEM. ^**^ *p* < 0.01, two‐tailed unpaired *t*‐test. L) Kaplan–Meier curves of survival for different subtypes of patients. Censored data is represented by vertical lines in the graph. *P* value is calculated using the log‐rank test. M) The bar chart represents the number of cases from patients who received standard chemoradiotherapy and had an outcome of death in MES and PN subtypes. N) Kaplan–Meier curves of survival for patients selected in M), stratified by subtype in GBM TMAs. *P* value is calculated using the log‐rank test.

We further investigated the role of MSN in MES‐subtype GBM. First, using the TCGA GBM database, we observed that MSN was highly expressed in the MES‐subtype GBM, and minimally expressed in the PN‐subtype (**Figure** **6**A). Moreover, the high MSN group was significantly enriched in the VERHAAK_MES_2017 pathway, whereas the low MSN group was enriched in the VERHAAK_PN_2017 pathway (Figure 6B,C).^[^ ^8^ ^]^ Additionally, in patients with GBM, correlation analysis revealed that MSN expression was positively correlated with MES pathway‐related and marker genes (CD44, YKL40, TIMP1, and TGFB1), whereas it was significantly negatively correlated with PN pathway‐related and marker genes (OLIG2, DLL3, SOX2, ASCL1) (Figure 6D).^[^ ^28^, ^29^, ^30^ ^]^ In summary, these results indicate that MSN contributes to the promotion of PMT. We then examined the expression of MSN, MES, and PN markers in GSCs. Consistent with previous observations, the results showed that MSN is highly expressed in MES GSCs (Figure 6E). To further explore whether MSN influences PMT, we designed a CD44 (MES marker) reporter system to monitor changes in the state of MES GSCs (Figure 6F). The results showed that MSN knockdown in MES GSCs led to a decrease in MES marker gene expression, but an increase in the expression of PN marker genes (Figure 6G,H; Figure S7A,B, Supporting Information). Additionally, the key transcriptional regulators of the MES subtype, such as, CEBPB, NFKB, STAT3, and YAP/TAZ signaling, were also weakened (Figure 6I). These data suggest that MSN may promote the PMT process by regulating key transcriptional regulators of the MES subtype.

Subsequently, we performed a multiplex immunofluorescence analysis of TMAs from patients with GBM. After excluding low‐quality spots, we classified the patients into MES and PN subtypes based on the expression of MES and PN marker genes (Figure S7C, Supporting Information). Combined with an immunohistochemical analysis of MSN expression, we observed that MSN was highly expressed in the MES subtype of GBM and was associated with poorer prognosis in these patients (Figure 6J–L). Finally, we conducted a survival analysis of patients with MES or PN subtype GBM who received standard chemoradiotherapy, and for whom the outcome was death. The results showed that patients with the MES subtype and high MSN expression had poorer survival outcomes and were more resistant to chemoradiotherapy than patients with the PN subtype and low MSN expression (Figure 6M,N). Collectively, these in vitro and clinical findings strongly suggest that MSN enhances chemoradiotherapy resistance in GBM by promoting PMT, highlighting MSN as a potential therapeutic target for MES subtype GBM.

### 2.5 GNE‐317 Enhances Sensitivity to RT and TMZ in GSCs by Reversing PMT via MSN Inhibition

**Figure 7:**
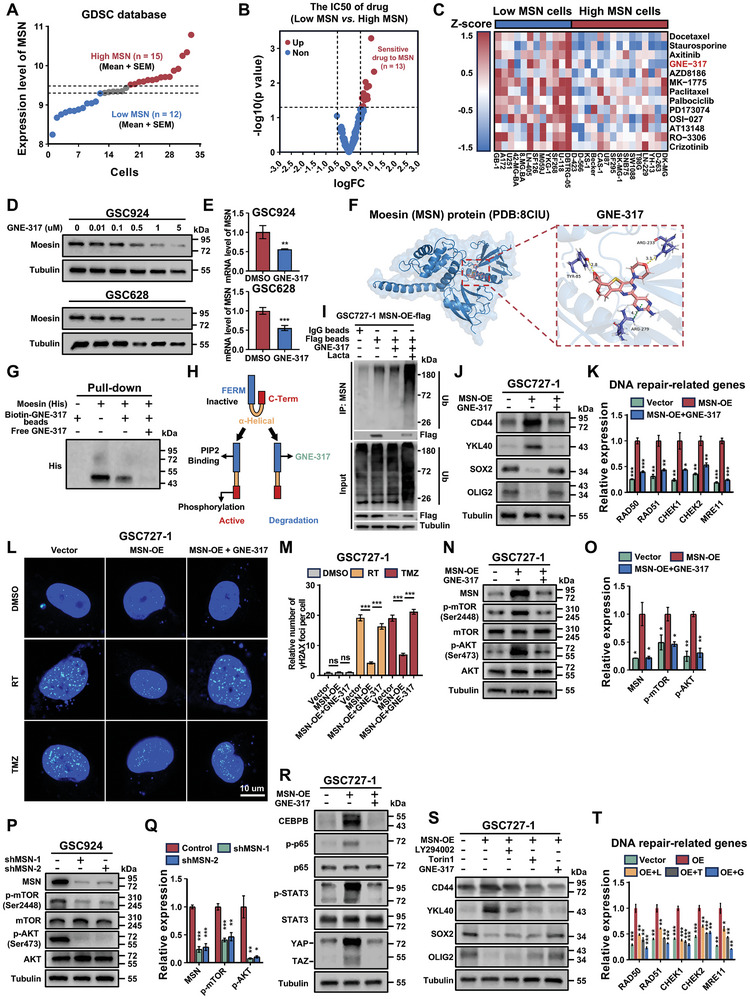
GNE‐317 enhances sensitivity to RT and TMZ by inhibiting MSN in GSCs. A) The scatter plot shows 33 glioma cells divided into high‐MSN (Mean + SEM) and low‐MSN (Mean – SEM) groups based on MSN expression levels. B) The volcano plot shows differential IC50 values of 141 drugs between high‐MSN and low‐MSN group cells. Red dots represent drugs sensitive to MSN. The x‐axis and y‐axis represent log_2_ (fold change) and –log_10_ (*p* value), respectively. C) The heatmap shows the relative IC50 value of 13 drugs sensitive to MSN in high MSN and low MSN groups. Red drug indicates the ability to cross the blood‐brain barrier. D) Immunoblot and E) qPCR analyses of the inhibitory effect of different concentrations of GNE‐317 on MSN at protein and RNA level in GSCs. F) Molecular docking analysis (3D Binding model) of Moesin protein (PDB:8CIU) with GEN‐317. GEN‐317 is shown as brick red sticks. The key residues are shown as blue sticks. Hydrogen bonds are shown as yellow dashed lines. Cation‐Pi interaction is shown as a green dashed line. G) Pull‐down assay demonstrates the protein interaction between GNE‐317 and the recombinant N‐terminal FERM domain of Moesin. Biotin‐GNE‐317 beads. Biotin‐GNE‐317 beads were constructed by conjugating biotinylated GNE‐317 to streptavidin‐coated beads. Free GNE‐317 refers to the addition of unbound GNE‐317 to recombinant of Moesin prior to pull‐down assays to neutralize binding. H) Hypothetical schematic diagram of Moesin activation under normal conditions and its inhibition by GNE‐317. I) Immunoprecipitation (IP) and immunoblot (IB) analyses of MSN polyubiquitination in GSC727‐1 overexpressing Flag‐tagged MSN, with or without GNE‐317 treatment. GSC727‐1 were then treated with 10 µM lactacystin (lacta) for 5h. Ub, ubiquitin. J) Immunoblot analysis of subtype marker genes in GSC727‐1 (Vector or MSN overexpression) treated with GNE‐317 (5 µm). K) Relative mRNA levels of DNA repair‐related genes in GSC727‐1 (Vector, MSN‐OE, MSN‐OE+GNE‐317 (5 µm)) with or without RT (6 Gy, 48 h) or TMZ (200 µm, 48 h) treatment. Data are shown as mean ± SEM. *n* = 3 independent experiments. ^*^ *p* < 0.05, ^**^ *p* < 0.01, ^***^ *p* < 0.001; two‐tailed unpaired *t*‐test. L) Representative images and M) quantification of γ‐H2AX staining on GSC727‐1 (vector, MSN‐OE, MSN‐OE+GNE‐317) with or without RT treatment (6 Gy, 48 h) or TMZ (200 µm, 48 h). Scale bars, 10 µm. Data are represented as means ± SEM. *n* = 5 independent experiments. ^***^ *p* < 0.001, ns, *p* >0.05; two‐tailed unpaired *t*‐test. N) Immunoblot and O) quantification analysis of downstream PI3K/mTOR signaling pathway in GSC727‐1 (Vector or MSN overexpression) treated with GNE‐317 (5 µm). P) Immunoblot and Q) quantification analysis of downstream PI3K/mTOR signaling pathway in GSC924 transduced with shNT or shMSN. R) Immunoblot analysis of key MES transcriptional regulators and pathways in GSC727‐1 (vector control or MSN overexpression) treated with GNE‐317 (5 µm). S) Immunoblot analysis of subtype marker genes in GSC727‐1 cells (vector control or MSN overexpression) treated with the PI3K inhibitor (LY294002, 10µm), the mTOR inhibitor (Torin1, 10 nm), or GNE‐317 (5 µM). T) Relative mRNA levels of DNA repair‐related genes in GSC727‐1 (Vector, MSN‐OE, MSN‐OE+LY294002 (10µm), MSN‐OE+Torin1 (10 nm), MSN‐OE+GNE‐317 (5 µm)). Data are shown as mean ± SEM. *n* = 3 independent experiments. ^*^ *p* < 0.05, ^**^ *p* < 0.01, ^***^ *p* < 0.001; two‐tailed unpaired *t*‐test.

Recent advancements in computer‐aided drug design have accelerated drug discovery, reducing both time and cost.^[^ ^31^ ^]^ Using high‐throughput screening methods, we identified 13 small‐molecule drugs that targeted MSN‐expressing cells in GBM (**Figure** **7**A,B). However, among these small‐molecule drugs, only GNE‐317 has been validated to cross the BBB (Figure 7C).^[^ ^32^ ^]^ Analysis of the TCGA GBM database suggests that MSN may influence chemoradiotherapy resistance via the PI3K/mTOR signaling pathway (Figure S8A–E, Supporting Information). Interestingly, GNE‐317 is also a specific inhibitor of the PI3K/mTOR pathway. Importantly, the inhibition of MSN in GSCs by GNE‐317 occurs primarily at the protein level, with minimal effects observed at the RNA level (GNE‐317: 5 µM) (Figure 7D,E). Moesin protein is a member of the ERM (Ezrin‐Radixin‐Moesin) protein family. Its structure is composed of three main parts: an N‐terminal FERM domain, a C‐terminal domain, and an α‐helical region connecting the two.^[^ ^33^, ^34^ ^]^ In its inactive state, moesin adopts a “closed” conformation, where the FERM domain and the C‐terminal domain interact via a head‐to‐tail interaction. When PIP_2_ binds to the FERM domain, it disrupts the “closed” conformation, exposing the C‐terminal domain. This exposure allows phosphorylation of a threonine residue in the C‐terminal domain. These two events together lead to the activation of moesin, enabling it to perform its functions.^[^ ^35^, ^36^, ^37^ ^]^ Furthermore, molecular docking analysis revealed that the GNE‐317 directly binds to the FERM domain of moesin protein (aa:1‐296) (Figure 7F). Therefore, leveraging the amino (‐NH2) group in the molecular structure of GNE‐317, we constructed biotin‐GNE‐317 beads (Figure S8F, Supporting Information). In subsequent pull‐down assays, we found that the biotin‐GNE‐317 beads could bind to the moesin N‐terminal recombinant protein (aa:1–346), and this binding was competitively inhibited by the prior addition of free GNE‐317 (Figure 7G). The above results demonstrate that GNE‐317 can directly bind to the FERM domain of the moesin protein. Based on the above results, we speculate that when GNE‐317 replaces PIP_2_ in binding to the FERM domain of the moesin protein, moesin may become an open but inactive abnormal protein. This abnormal protein might be recognized by the body and degraded (Figure 7H). Furthermore, we observed that polyubiquitinated moesin decreased following treatment with GNE‐317 but increased significantly upon the addition of the proteasome inhibitor lactacystin (Figure 7I). This suggests that the abnormal moesin protein bound to GNE‐317 is degraded through ubiquitin‐mediated proteolysis. Therefore, we selected GNE‐317 as the drug to target MSN in GBM. We subsequently found that MSN overexpression in PN GSCs (GSC727‐1) promoted PMT and enhanced DNA damage repair following RT and TMZ treatment, thereby driving chemoradiotherapy resistance (Figure 7J–M). Furthermore, MSN was found to independently induce AKT and mTOR phosphorylation, activating the PI3K/mTOR pathway without reliance on GNE‐317 (Figure 7N–Q). However, treatment with GNE‐317 reversed these effects in GSC727‐1 (Figure 7J–O) and suppressed the activation of the key transcriptional regulators of the MES subtype driven by MSN overexpression (Figure 7R). Finally, we found that solely inhibiting the PI3K or mTOR signaling pathway could partially suppress the PMT process and weaken DNA damage repair. However, its effectiveness is limited compared to dual inhibitors like GNE‐317 (Figures S‐T).

**Figure 8:**
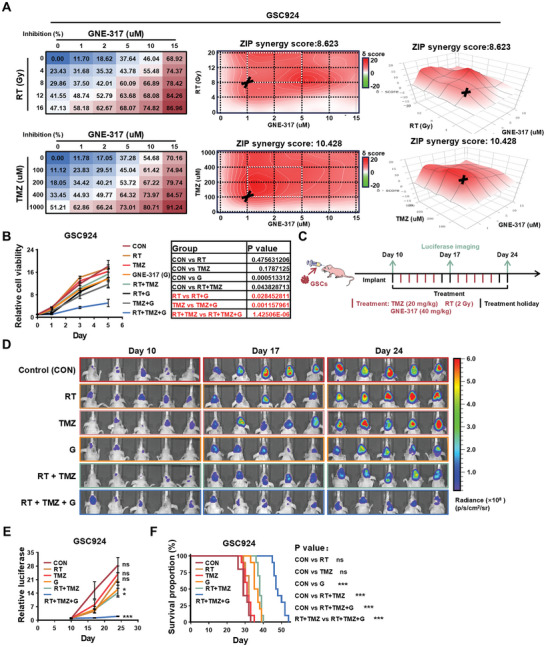
GNE‐317 can reduce resistance to RT or TMZ in GSCs. With the indicated concentrations of GEN‐317 and RT/TMZ for 48 h. A) Cell inhibition matrices (left), synergy scores (middle), and 3D ZIP model (right) were calculated using the ZIP model in GSC924. White boxes and black crosses mark the optimal combination. B) Cell viability assay of GSCs treated with different treatments (RT: 8 Gy, TMZ: 100 µM, GNE‐317: 1 µm). *n* = 6 biological independent samples. Data are shown as means ± SD. *P* values are in the table on the right, two‐way ANOVA analysis followed by Tukey's multiple test. C) A schematic of the experimental procedure for the GSC924 orthotopic xenograft model. Tumor‐bearing nude mice were treated with specified methods (DMSO (CON), RT, TMZ, GNE‐317, RT +TMZ, and RT +TMZ + GNE‐317) by gavage for 2 weeks, respectively. D) Representative images and E) quantification analysis of relative luciferase on days 10, 17, and 24 are shown. *n* = 5 for each group. Data are represented as means ± SEM. ^*^ *p* < 0.05, ^***^ *p* < 0.001, ns, *p* > 0.05; one‐way ANOVA with Tukey's method for multiple comparisons. F) Kaplan–Meier survival curves of mice bearing GSC924‐derived xenografts treated with different treatments. ^***^ *p* < 0.001, ns, *p* > 0.05; log‐rank test. *n* = 10 biological independent samples.

We also investigated whether GNE‐317 exhibits a synergistic effect with the first‐line treatments RT and TMZ in MES GSCs. Combination matrices of cell inhibition and synergy scores (based on the ZIP model) demonstrated strong synergy in GSC924 cells with the combination treatment (**Figure** **8**A). Considering optimal inhibitory efficiency and drug toxicity, we selected RT 8 Gy with GNE‐317 1 µm and TMZ 100 µm with GNE‐317 1 µm as the optimal combinations. In vitro, cell viability assays further demonstrated that the combined treatment not only exhibited stronger growth inhibitory effects but also significantly enhanced the sensitivity of cells to RT and TMZ (Figure 8B). Given that the first‐line clinical treatment for GBM is a combination of RT and TMZ, with RT or TMZ rarely used alone, we designed in vivo experiments using only a combined RT and TMZ treatment group (TMZ: 20 mg^−1^kg, RT: 2 Gy, GNE‐317: 40 mg^−1^kg, administered five times per week for two consecutive weeks) (Figure 8C).^[^ ^38^ ^]^ Our results showed that the addition of GNE‐317 to the standard RT and TMZ treatment led to a notable inhibition of GSC growth in vivo and significantly prolonged mouse survival (Figure 8D–F). In conclusion, GNE‐317 exhibited robust synergy with RT and TMZ, markedly enhancing the sensitivity of MES GSCs to RT and TMZ. These findings suggest that the combination of GNE‐317 and standard chemoradiotherapy offers a promising new therapeutic strategy for improving outcomes in patients with GBM.

## 3 Discussion

GBM is a highly aggressive brain tumor with a poor prognosis, primarily due to its resistance to standard first‐line therapies such as RT and TMZ. This resistance leads to recurrence and poses challenges for long‐term treatment. In this study, we identified MSN as a key driver promoting the PMT process in GSCs by activating the PI3K/mTOR signaling pathway. The resulting MES GSCs exhibit resistance to chemoradiotherapy by enhancing DNA damage repair following RT and TMZ treatments, thereby sustaining GBM malignancy and promoting tumor recurrence.

Moesin (MSN), a membrane‐cytoskeletal linker protein, is critical for maintaining cell cortex organization and plays a pivotal role in cell morphogenesis and differentiation.^[^ ^35^ ^]^ Numerous studies have demonstrated that moesin is overexpressed in various tumors and plays a crucial role in their progression.^[^ ^39^, ^40^ ^]^ Moesin is suggested to be a potential marker for epithelial‐mesenchymal transition (EMT) in both breast and pancreatic cancers.^[^ ^41^, ^42^, ^43^ ^]^ In addition, Moesin has been found to activate the Wnt/β‐catenin pathway and the PI3K/mTOR signaling pathway, serving as a marker for glioma progression.^[^ ^44^ ^]^ This provides theoretical support for the finding that MSN can activate the PI3K/mTOR signaling pathway in our study. Then, through a comprehensive analysis of the Cancer Cell Line Encyclopedia (CCLE) and Genomics of Drug Sensitivity in Cancer (GDSC) databases, we identified GNE‐317, a PI3K/mTOR inhibitor that crosses the blood‐brain barrier and specifically targets Moesin. By reversing the MSN‐Mediated PMT process, GNE‐317 synergizes with RT and TMZ to significantly enhance the sensitivity of GBM cells to chemoradiotherapy. (Figure S9, Supporting Information).

RT and TMZ are standard first‐line treatments for patients with GBM. TMZ is an alkylating agent that induces DNA damage by adding methyl groups to guanine at the O6 position, leading to DNA mismatches, persistent DNA double‐strand breaks (DSBs), and ultimately cell death.^[^ ^45^, ^46^ ^]^ Meanwhile, RT generates DNA damage primarily through ionizing radiation, leading to the formation of DSBs, which, if unrepaired, can trigger cell death.^[^ ^47^, ^48^ ^]^ However, extensive research has shown that glioma stem cells (GSCs) contribute significantly to resistance against both TMZ and RT.^[^ ^49^, ^50^ ^]^ On the one hand, GSCs possess strong tumorigenic potential and the ability to maintain stemness, which supports continued tumor growth and therapy resistance.^[^ ^51^ ^]^ On the other hand, the PMT in GSCs is considered a hallmark of GBM recurrence and multi‐therapy resistance.

The mechanisms driving PMT have drawn considerable interest. For instance, it has been shown that this PMT is mediated by C/EBPβ activation in GSCs after RT. Other studies have suggested that suppressing ASCL1 expression or elevating NDRG1 level in PN‐GSCs can likewise trigger PMT, further contributing to chemoradiotherapy resistance.^[^ ^15^ ^]^ Additionally, the tumor microenvironment (TME) contributes to PMT, with macrophage‐derived factors, such as oncostatin M (OSM), promoting the MES state and resistance to chemoradiotherapy.^[^ ^52^ ^]^ Moreover, our previous research indicated that CEBPB+ GBM subclusters can promote M2 macrophage polarization and that these M2 macrophages further contribute to the malignant progression of MES GBM cells, thereby enhancing their resistance to chemoradiotherapy.^[^ ^22^ ^]^ Together, these findings suggest that PMT is driven by a multifaceted network of intrinsic and extrinsic factors. In this study, we found that MSN can activate the PI3K/mTOR signaling pathway in GSCs, thereby upregulating the expression of key transcription factor networks and signaling pathways of the MES subtype (including CEBPB, NFKB, STAT3, and YAP/TAZ signaling), which triggers the PMT process and leads to resistance to RT and TMZ both in vitro and in vivo. This discovery provides novel insights into the mechanisms underlying PMT and highlights the potential of MSN as a therapeutic target to overcome treatment resistance in GBM.

We focused our efforts on addressing chemoradiotherapy resistance in GBM by using adjuvant pharmacotherapy, specifically through repurposing existing small‐molecule drug inhibitors, for several key reasons. First, the presence of GSCs, particularly MES GSCs, drives GBM heterogeneity and resistance to treatment. Small‐molecule inhibitors can specifically target pathways underlying MES GSC therapy resistance, such as enhanced DNA repair. Second, GBM does not appear to be driven by a single pathway, as demonstrated by the lack of success in most monotherapy‐based clinical trials for this malignancy.^[^ ^53^ ^]^ By combining small‐molecule inhibitors with existing treatments such as RT and TMZ, it is possible to achieve synergistic effects that enhance therapeutic efficacy and overcome resistance. Third, reversing the PMT process with small‐molecule drugs capable of crossing the BBB to re‐sensitize GBM to chemoradiotherapy is a highly feasible therapeutic strategy. In this study, we identified GNE‐317, a small molecule capable of targeting MSN and reversing the PMT process. However, previous studies have shown that GNE‐317 exhibits varying inhibitory effects across different GBM cell lines. For example, while Chani et al. reported no significant effect on GL261 cells, Laurent et al. found that GNE‐317 notably inhibited the growth of U87, GS2, and GBM10 cells.^[^ ^38^, ^54^ ^]^ These findings suggest that GNE‐317 may have specific targeting effects, consistent with our data showing that GNE‐317 had a significantly lower IC50 in high MSN GBM cells, such as U87 and T98G cells, which are typically highly resistant to chemoradiotherapy (Figure 1D and 7C). This further demonstrates that GNE‐317 may serve as a specific inhibitor of the MES subtype with high MSN expression to enhance GBM sensitivity to chemoradiotherapy.

## 4 Conclusion

Our investigation revealed MSN functions as a crucial regulator of PMT in GSCs, driving resistance to RT and TMZ in GBM. We further demonstrated that GNE‐317 specifically targets MES GSCs with high MSN expression, reversing the PMT process and resensitizing these cells to chemoradiotherapy. Our analysis also showed that the variability in GNE‐317′s effects across GBM cell lines is likely due to differences in MSN expression. Specifically, GNE‐317 demonstrated a lower IC50 value in MES GSCs with high MSN expression. These findings underscore the potential of GNE‐317 as a targeted inhibitor of MES in GBM and offer an encouraging therapeutic strategy to overcome chemoradiotherapy resistance and enhance treatment outcomes for patients with GBM.

## 5 Experimental Section

### Data Accessibility

Bulk RNA‐sequencing data (TCGA, CGGA, Rembrandt, and Ivy GAP databases) were obtained from GlioVis (http://gliovis.bioinfo.cnio.es/). The Gene Expression Omnibus (GEO, http://www.ncbi.nlm.nih.gov/geo/) provided the scRNA‐seq data for glioma (GSE117891), and spatial transcriptomics (ST) data were obtained from the 10X Genomics platform (https://www.10xgenomics.com/cn). Drug sensitivity data for GBM cell lines were retrieved from the Genomics of Drug Sensitivity in Cancer (GDSC, www.cancerRxgene.org), while transcriptional profiles for these cell lines were obtained from the Cancer Cell Line Encyclopedia (CCLE, https://portals.broadinstitute.org/ccle/).

### scRNA‐Seq Data and Spatial Transcriptomics Data Process

scRNA‐seq and ST data were analyzed utilizing the R package Seurat (version 4.1.0). For scRNA‐seq, the R package DoubletFinder was first used to remove non‐single cells based on the optimal pK value, then filtered out low‐quality cells with fewer than 200 total feature RNAs and with genes expressed in fewer than three cells. Dimensional reduction was conducted through principal component analysis (PCA) (npcs = 30), succeeded by t‐distributed stochastic neighbor embedding (tSNE) algorithmic processing (dims = 1:10). Initial analysis utilizing FindNeighbors and FindClusters functions revealed nineteen distinct cell groupings. Characteristic genes were identified in each grouping through the FindAllMarkers function (only.pos = TRUE, min.pct = 0.25 and logfc.threshold = 0.5). Further, each cluster was annotated manually using CNV values predicted by the inferCNV package (version 1.8.0) along with marker genes. CNV calculations for all cell categories were derived from single‐cell sequencing data, implementing a 0 cutoff and 0.2 noise filter. Expression data underwent re‐standardization per sample, constraining values between ‐1 and 1. Each cell's CNV score was ascertained through quadratic summation of CNV regions.^[^ ^55^ ^]^ Non‐tumor cells and glioma cells were then further subdivided into 10 and 14 subclusters, respectively. Spatial transcriptomics data were processed according to the developer's recommended guidelines (https://satijalab.org/seurat/articles/spatial_vignette.html). Finally, data normalization was performed using the SCTranform function, followed by dimensionality reduction using principal component analysis and tSNE algorithms. Clustering was achieved using the default resolutions of the first 30 principal components.

### Integrating scRNA‐Seq and Spatial Transcriptomic Data

A joint analysis of scRNA‐seq and spatial transcriptomic data was performed using the integration features provided by the Seurat package. First, dimensionality reduction was preprocessed and performed on the scRNA‐seq and spatial transcriptomic data as described above. The FindTransferAnchors function was then used to identify anchors between scRNA‐seq and spatial transcriptomic data (normalization.method = “SCT”), and apply the TransferData function to calculate prediction scores between them (dim = 1:30). Finally, the prediction scores of different single‐cell subclusters were obtained across various spatial clusters.

### Gene Set Enrichment Analysis (GSEA) and Pathway Analysis

First, the Limma package was used to perform differential gene expression analysis between the high and low‐MSN groups. The 296 genes highly regulated were then used by MSN (Log_2_ (fold change) > 0.5, ‐Log_10_ (*p* value) > 10) for the gene set enrichment analysis (GSEA software, version 4.1.0); pathway enrichment analysis was executed utilizing the R package clusterProfiler (version 4.0.5).

### Molecular Docking

A molecular docking model of Moesin was established with GNE‐317. The molecular structure of moesin was obtained from the RCSB Protein Data Bank (http://www.rcsb.org) and processed using Schrödinger Protein Preparation Wizard (https://www.schrodinger.com). The LigPrep module with the OPLS3e force field was utilized to perform ionization and minimization of GNE‐317′s structure. The preprocessed moesin and GNE‐317 underwent standard precision docking through the Glide module in the ligand‐docking component.

### Cell and Culture Condition

All cells were kept in a moisture‐controlled chamber at 37 °C under 5% CO_2_ conditions. Commercial ATCC lines (NHA, A172, A1207, LN18, T98G, U251, and LN229) were grown in DMEM (Gibco) enriched with 10% fetal bovine serum (Mei5bio) and 1% penicillin‐streptomycin (Bioss). Glioblastoma tissue from GSCs (GSC428‐1, GSC428‐4, PGSC8, GSC3, GSC924, and GSC628) originated from surgical resections of glioblastoma patients treated at the Department of Neuro‐Oncology and Neurosurgery within Tianjin Medical University Cancer Institute and Hospital, Tianjin, China. The ethics board of Tianjin Medical University Cancer Institute and Hospital authorized all human tissue protocols (Ek2023099). Each participant provided written consent. Patient‐derived xenograft models served as sustainable GSC sources. STR profiling confirmed the authenticity of tumor models. The GSC populations were expanded as spheroid formations in neurobasal medium (Gibco) containing 1% B27 supplement (Gibco), 2 mm L ‐glutamine, 1 mm sodium pyruvate, along with 10 ng‐1 mL bFGF and EGF (Gibco).

### Realtime‐qPCR (RT‐qPCR) Analysis

Total mRNA was procured and cleaned utilizing an RNA isolation kit (SparkJade) following the protocol provided by the manufacturer. An amount of 500 ng of messenger RNA underwent reverse transcription to generate cDNA employing the UEIris RT mixture with DNase components (Us EVERBRIGHT) in a T20 thermocycler (LongGene). RT‐qPCR analysis was performed utilizing Universal SYBR Green qPCR Supermix (Us EVERBRIGHT) components in a 7900‐thermocycler system (Applied Biosystems). The cycling protocol consisted of 32 cycle rounds with three phases: 95 °C lasting 30 s, 60 °C lasting 10 s, and 72 °C lasting 30 s. Expression values were normalized to 18S, and each RT‐qPCR was performed in triplicate for data analysis. Gene‐specific primers are listed in Table S1 (Supporting Information).

### Immunoblot Analysis

Harvested cells underwent lysis utilizing RIPA buffer (Thermo Scientific) supplemented with phosSTOP phosphatase inhibitor (Roche) and protease inhibitor mixture (Sigma). Protein separation was accomplished through SDS‐PAGE (NuPAGE Bis‐Tris gel, Invitrogen) followed by NC membranes (Millipore) transfer. The membranes underwent blocking in 5% (wt/vol) non‐fat milk dissolved in TBS containing 0.5% (vol/vol) Tween‐20, followed by incubation with primary antibodies targeting Tubulin (1:5000, EASYBIO), MSN (1:5000, Abcam), γ‐H2AX (1:1000, CST), TP53 (1:1000, CST), Caspase‐3 (1:1000, Proteintech), AKT (1:2000, ABclonal), p‐ATK (1:1000, ABclonal), mTOR (1:2000, ABclonal), p‐mTOR (1:1000, ABclonal), YKL40 (1:2000, Abcam), CD44 (1:2000, Proteintech), SOX2 (1:1000, Proteintech), OLIG2 (1:2000, Abcam), Ubiquitin (1:1000, ABclonal), CEBPB (1:1000, Santa cruz), NFKB p65 (1:5000, ABclonal), p‐NFKB p65 (1:10000, ABclonal), STAT3 (1:2000, ABclonal), p‐STAT3 (1:10000, ABclonal), YAP/TAZ (1:1000, CST) at 4 °C overnight. Following triple TBST washes, the membranes underwent room temperature incubation with HRP‐conjugated secondary antibodies: anti‐mouse IgG (CST, 7076), anti‐rabbit IgG (CST, 7074), and anti‐goat IgG (EASYBIO, BE0103) in 5% milk for 1 h. Signal detection employed luminol reagent (Millipore, WBKLS), with image acquisition performed using a molecular imager (BLT PHOTON TECHNOLOGY, GV6000 PLUS) and subsequent analysis via GV6000 M2 software. For the quantitative analysis of immunoblots, the Gels tool was used in ImageJ software to analyze bands. Three independent biological experiments were performed, and the quantified values were subjected to statistical analysis.

### Human Tissue Microarray (TMA) Immunostaining and Multiplexed Immunofluorescence Assays

The TMA consisted of 101 tissue spots. Among these, 27 spots were derived from normal brain tissues obtained from autopsy specimens. The remaining spots consisted of glioma samples (WHO grades 1–4) collected from patients at Tianjin Medical University Cancer Institute and Hospital. Ethical approval for all procedures involving using human tissues was granted by the ethics committee of Tianjin Medical University Cancer Institute and Hospital (Ek2023099). Furthermore, informed consent was secured from all tissue donors. For immunostaining, the MSN antibody (1:50, Abcam) was employed to detect signals. A semi‐quantitative evaluation of tissue staining was performed by converting the proportion of positive cells and their staining intensities into corresponding numerical values. The H‐Score was determined using the following formula: H‐Score = ∑(pi × i) = (percentage of weak intensity area × 100) + (percentage of moderate intensity area × 200) + (percentage of strong intensity area × 300).^[^ ^56^ ^]^ To identify the molecular subtype of each patient's GBM, FFPE slides of the tissue samples underwent multiplexed immunofluorescence and multispectral imaging using a PANO Multiplex IHC kit (Panovue), targeting CD44, YKL40, SOX2, and OLIG2. The staining procedure adhered to a standard protocol that entailed sequential incubation with primary and secondary antibodies (CD44: 1:8000, Abcam; YKL40: 1:1000, Abcam; SOX2: 1:400, Abcam; OLIG2: 1:500, Oasis Biofarm), followed by tyramide signal amplification (TSA). Nuclear staining was achieved using DAPI. Multispectral images were captured utilizing using the Mantra System (PerkinElmer). Tissue spots exhibiting detachment exceeding 30% were excluded from subsequent analysis.

### Cell Viability and Treatment

For the cell viability assay, the viability of cells was assessed on designated days following cell seeding, utilizing the Cell Counting Kit‐8 (TargetMol, USA, C0005) Assay Kit, in accordance with the manufacturer's instructions. GSCs (2000) were seeded into each well of a 96‐well plate, followed by incubation for 24 h for pre‐cultivation. Analysis was conducted on the specified days post‐seeding employing an enzyme‐linked immunosorbent assay reader. For treatments, TMZ and GNE‐317 were dissolved in dimethyl sulfoxide (DMSO), with the DMSO concentration strictly maintained below 0.1%. GSCs were exposed to varying drug and RT doses as dictated by the experimental protocol. At predetermined intervals, CCK‐8 assays were performed to evaluate the effects of the treatments.

### Tumorsphere Formation Assays and Limiting Dilution Assays

For the tumorsphere formation assay, 1000 GSCs were seeded into individual wells of a 96‐well plate. Tumorspheres were enumerated seven days post‐seeding. For the limiting dilution assays, GSCs were plated in a well of 96‐well plates at predefined densities (0, 1, 5, 10, 20, 50 cells), with 16 replicates for each density. After seven days, the presence and count of tumorspheres in each well were documented and analyzed using the ELDA software (http://bioinf.wehi.edu.au/software/elda/).

### Plasmid and Lentiviral Transduction

Lentiviral plasmids for MSN shRNA knockdown (shMSN‐1, shMSN‐2), MSN overexpression, and nonspecific control sequences (CON054) were obtained from Genechem (Shanghai, China). The vector elements hU6‐MCS‐CMV‐Puromycin were utilized for MSN shRNA knockdown, while Ubi‐MCS‐3FLAG‐SV40‐BSD was employed for MSN overexpression. Lentiviral particles were generated in 293T cells through the use of PAX2 and PMD2G helper plasmids (Addgene) in DMEM. For lentiviral transduction, GBM cells were infected with lentiviruses expressing the shMSN, MSN overexpression, or control sequences for 48 h before being subjected to subsequent analysis. For the CD44 reporter system, two luciferase constructs were transfected into the GSCs. Firefly‐luciferase, driven by the CMV promoter, served as an internal control, while NanoLuc‐luciferase driven by the CD44 promoter was used to assess CD44 expression levels. After transfection, fluorescence signals were detected separately using substrates specific for F‐luciferase or N‐luciferase.

### DNA Damage Detection

To evaluate DNA damage in cells following RT and TMZ treatments, a γ‐H2AX immunofluorescence assay was conducted. Cells were fixed using 4% paraformaldehyde for 15 min at room temperature and subsequently permeabilized with 0.1% Triton X‐100 for 10 min. Following PBS washes, blocking was performed with 5% BSA for 1 h. Cells were then incubated overnight at 4 °C with a primary antibody targeting γ‐H2AX (1:200, CST). On the following day, the cells were washed and treated with a fluorescently labeled secondary antibody for 1 h at room temperature. Nuclei were counterstained with DAPI. Confocal microscopy (Zeiss, LSM 880) was utilized to capture images and assess DNA damage. Quantification of γH2AX foci was performed using the ImageJ software. Immunofluorescence images were first processed to enhance contrast and remove background noise. Individual γH2AX foci were identified and counted using the “Analyze Particles” function in ImageJ after appropriate thresholding. For each group, 5 randomly selected images were analyzed to determine the number of γH2AX foci per cell. Quantified values were subjected to statistical analysis.

### Animal Experiments and Treatment

All animal procedures were sanctioned by approval from the Animal Ethics and Welfare Committee of the Tianjin Medical University Cancer Institute and Hospital, Tianjin, China (Approval No. AE‐2022111, Ek2020157). Four‐week‐old female nude mice obtained from Beijing SiPeiFu Biotechnology Co., Ltd, were utilized in these studies. The mice were housed under a controlled environment with a 12 h light/12 h dark cycle, a temperature range of 20–26 °C, and humidity maintained between 30–70%, and were provided with ad libitum access to food. For all in vivo animal models, the mice were first weighed, and selected those with similar body weights for the experiments. These mice were then randomly assigned to the experimental and control groups, followed by the orthotopic implantation of the corresponding GBM cells. The specific procedure was as follows: first, anesthesia was administered, and the mice were positioned on a stereotactic injection apparatus to facilitate tumor implantation surgery. A burr hole was drilled into the right cerebral cortex of each mouse after exposing the head. Firefly‐luciferase‐expressing GBM cells (GSC924 cells (2 × 10^5^) or GSC628 cells (2 × 10^5^)) were implanted into the right cerebral cortex of nude mice at a depth of 3.5 mm, and the incision was sutured. The size of the orthotopic tumor was monitored weekly via the bioluminescence channel of the IVIS Spectrum optical imaging system. The relative luciferase signal was quantified by comparing the luciferase signal at each detection time point for each mouse to its signal at the first detection, resulting in the relative luciferase signal to monitor tumor growth in vivo. For in vivo treatment, TMZ at a dose of 20 mg^−1^ kg was dissolved in a solvent composed of 40% polyethylene glycol‐300 (PEG‐300), 10% DMSO, 5% Tween‐80, and 45% saline. For intragastric administration, GNE‐317 at a dose of 40 mg^−1^ kg was prepared in a solution of 0.5% methylcellulose and 0.2% Tween‐80 (MCT). RT was administered using fractionated doses (2 Gy x 5) delivered through a ^60^Co teletherapy unit equipped with a custom gig and validated dosimetry. According to the standard protocol, treatments were performed five times per week.

### Drug Combination Assay

GSC924 was treated with varying concentrations and doses of treatments to investigate whether GNE‐317 exhibits a synergistic effect with RT or TMZ. The detailed procedure was as follows: First, an equal number of GSC cells were seeded in 96‐well plates. The cells were then treated with different concentrations of the drugs as follows: Group 1: RT (Gy): 0, 4, 8, 12, 16 and GNE‐317 (µm): 0, 1, 2, 5, 10, 15; Group 2: TMZ (µm): 0, 100, 200, 400, 1000 and GNE‐317 (µm): 0, 1, 2, 5, 10, 15. After 48 h of combined treatment, cell viability was assessed using the CCK8 assay. The results were normalized to the control group (untreated), and the inhibition rate of the drug combinations was calculated. Finally, the synergistic effect was quantified by calculating the synergistic score using the ZIP model in SynergyFinder based on the inhibition rates.^[^ ^57^ ^]^

### Statistical Analysis

All grouped data were presented as mean ± SEM or mean ± SD. Comparisons between groups were conducted using one‐way ANOVA or Student's *t*‐test. For Kaplan‐Meier survival analyses, statistical significance was determined via the Wilcoxon test or log‐rank test. To assess the correlation between samples, two‐sided Pearson correlation coefficients were employed to evaluate sample correlations. All statistical analyses were carried out using Microsoft Excel 2019, GraphPad Prism(versions 8 and 9), or R version 4.0.5, with statistical significance defined as *p* < 0.05 (^***^ *p* < 0.001; ^**^ *p* < 0.01; ^*^ *p* < 0.05; ns, *p* > 0.05). Additional details are provided in each figure legend. For all results, excluding those derived from the public databases, similar findings were obtained from three independent experiments.

## Conflict of Interest

The authors declare no conflict of interest.

## Supporting information



Supporting Information

## Data Availability

The data that support the findings of this study are available from the corresponding author upon reasonable request.
