# Supplementary material for: GNE‐317 Reverses MSN‐Mediated Proneural‐to‐Mesenchymal Transition and Suppresses Chemoradiotherapy Resistance in Glioblastoma via PI3K/mTOR
Source: Adv Sci (Weinh). 2025 Feb 7;12(12):2412517. doi: 10.1002/advs.202412517 (PMC11948001; doi:10.1002/advs.202412517)
Supplement: Supplementary file 1 — Supporting Information [file ADVS-12-2412517-s001.docx]

# Supporting Information

Supplementary Tables

**Table S1. Gene-specific primer information for qPCR.**

| Gene name | Primer sequence | |
| --- | --- | --- |
| 18S | forward | 5′-TGCATGGCCGTTCTTAGTTG-3′ |
|  | reverse | 5′-AGTTAGCATGCCAGAGTCTC-3′ |
| MSN | forward | 5′-CTGATGGAGAGGCTGAAGCAGA-3′ |
|  | reverse | 5′-ACGCTTCCGTTCCTGCTCAAGT-3′ |
| CD44 | forward | 5′-CCAGAAGGAACAGTGGTTTGGC-3′ |
|  | reverse | 5′-ACTGTCCTCTGGGCTTGGTGTT-3′ |
| YKL40 | forward | 5′-CCACAGTCCATAGAATCCTCGG-3′ |
|  | reverse | 5′-TGCCTGTCCTTCAGGTACTGCA-3′ |
| SOX2 | forward | 5′-GCTACAGCATGATGCAGGACCA-3′ |
|  | reverse | 5′-TCTGCGAGCTGGTCATGGAGTT-3′ |
| OLIG2 | forward | 5′-ATGCACGACCTCAACATCGCCA-3′ |
|  | reverse | 5′-ACCAGTCGCTTCATCTCCTCCA-3′ |
| RAD50 | forward | 5′-GGAAGAGCAGTTGTCCAGTTACG-3′ |
|  | reverse | 5′-GAGTAAACTGCTGTGGCTCCAG-3′ |
| RAD51 | forward | 5′-TCTCTGGCAGTGATGTCCTGGA-3′ |
|  | reverse | 5′-TAAAGGGCGGTGGCACTGTCTA-3′ |
| CHEK1 | forward | 5′-GTGTCAGAGTCTCCCAGTGGAT-3′ |
|  | reverse | 5′-GTTCTGGCTGAGAACTGGAGTAC-3 |
| CHEK2 | forward | 5′-GACCAAGAACCTGAGGAGCCTA-3′ |
|  | reverse | 5′-GGATCAGATGACAGCAGGAGTTC-3′ |
| MRE11 | forward | 5′-CAGCAACCAACAAAGGAAGAGGC-3′ |
|  | reverse | 5′-GAGTTCCTGCTACGGGTAGAAG-3′ |

Supplementary Figures


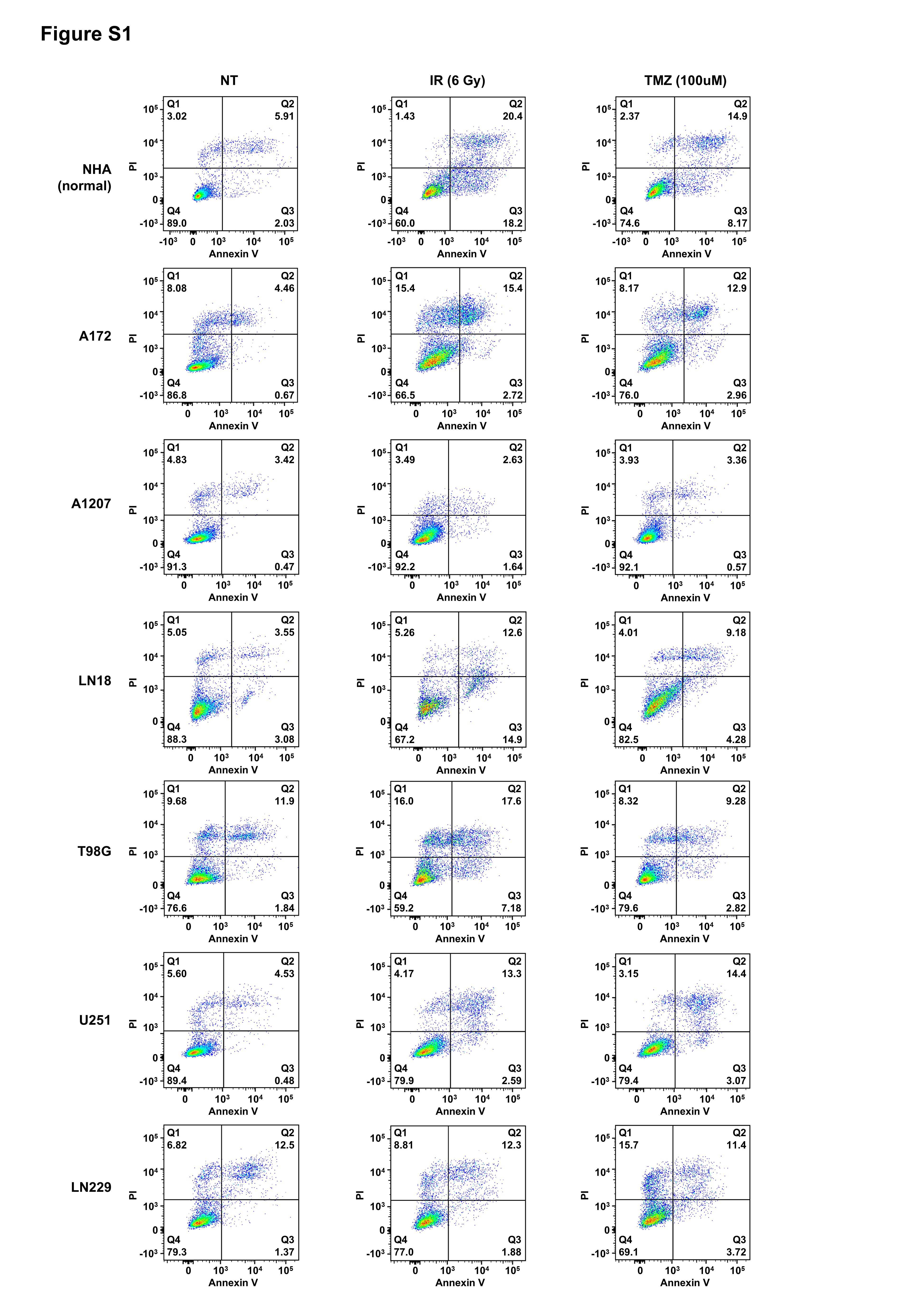


**Figure S1.** The apoptosis assay (Annexin V and PI) showing apoptosis levels in various cell lines after RT (6 Gy) and TMZ (100 μM) treatment.


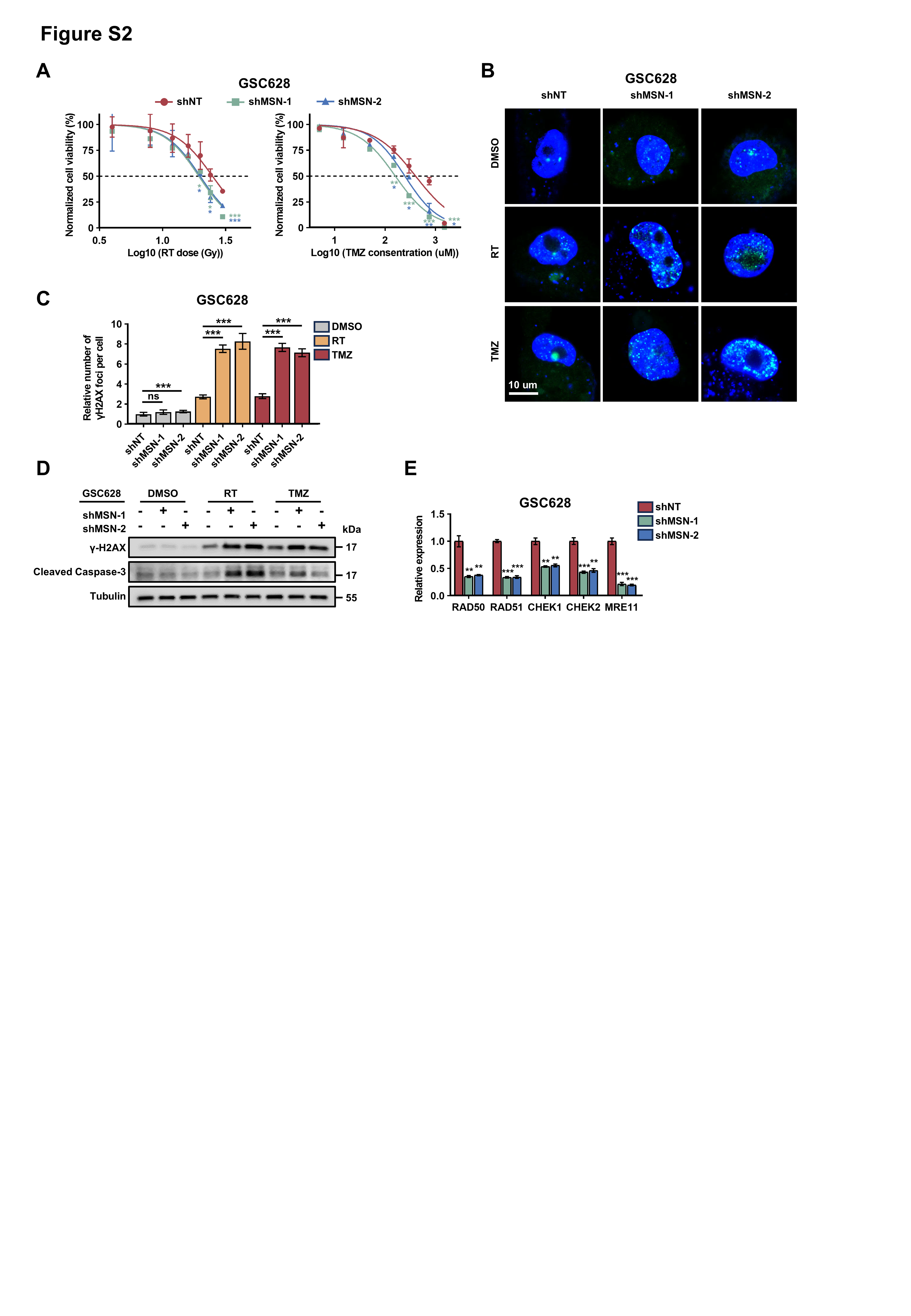


**Figure S2.** MSN enhances chemoradiotherapy resistance in glioma stem cells. A) Cell viability assay of GSC628 transduced with shNT or shMSN, treated with different doses of RT or varying concentrations of TMZ for 48 hours. n = 3 biological independent samples. The black dashed line represents the IC50 value. **p* < 0.02, ***p* < 0.01, ****p* < 0.001; two-way ANOVA followed by Tukey’s multiple comparison test. B) Representative images and C) quantification of γ-H2AX staining in GSC628 with or without RT (6 Gy, 48 h) or TMZ (200 μM, 48 h) treatment. Scale bar: 10 μm. Data are shown as mean ± SEM. n = 5 independent experiments. ****p* < 0.001, ns, *p* > 0.05; two-tailed unpaired t-test. D) Immunoblot analysis of γ-H2AX and cleaved Caspase-3 expression in GSC924 with or without RT (6 Gy, 48 h) or TMZ (200 μM, 48 h) treatment. E) Relative mRNA levels of DNA repair-related genes in GSC628 with or without RT (6 Gy, 48 h) or TMZ (200 μM, 48 h) treatment. Data are shown as mean ± SEM. n = 3 independent experiments. ***p* < 0.01, ****p* < 0.001; two-tailed unpaired t-test.


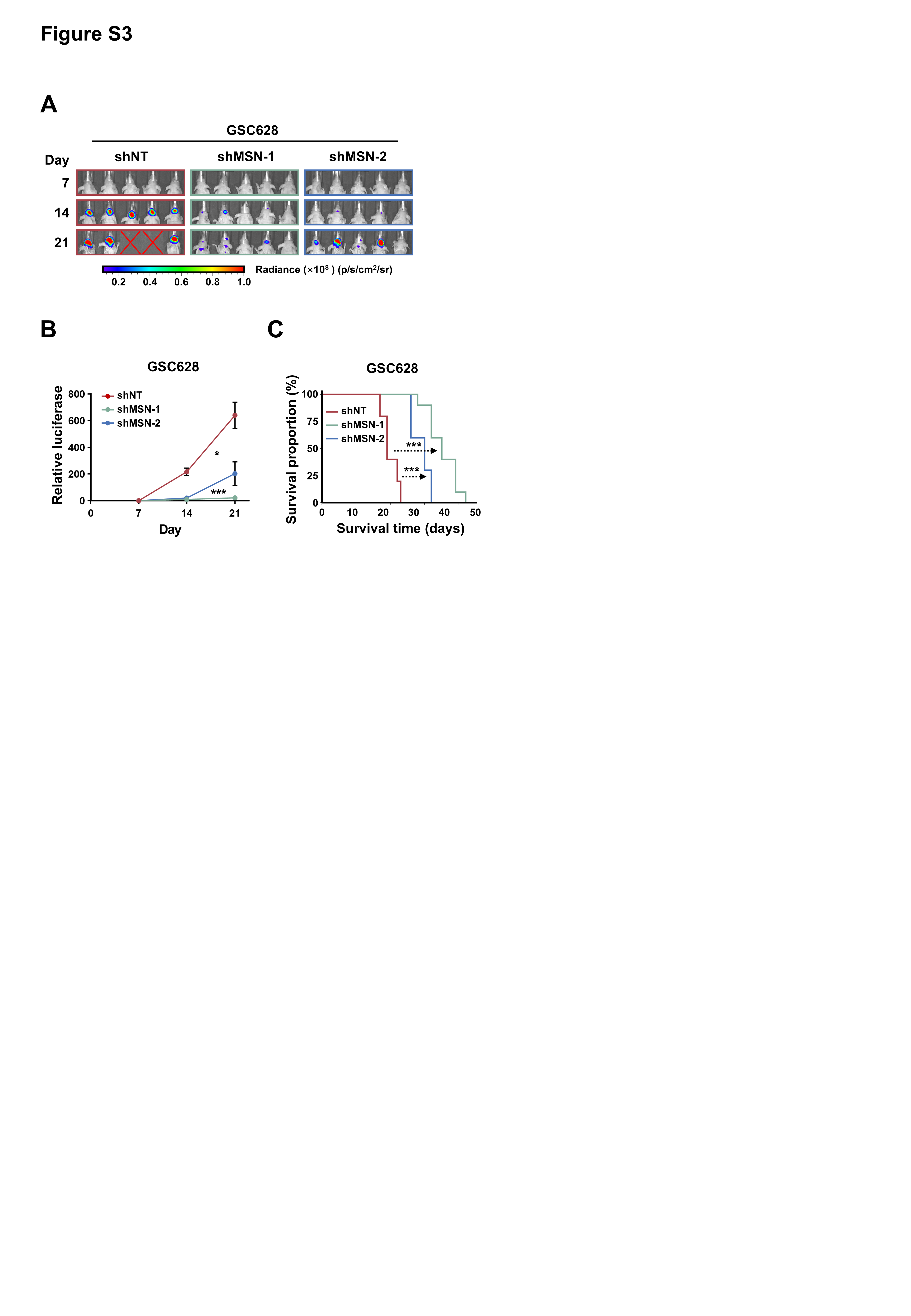


**Figure S3.** Disrupting MSN in GSCs suppressed GBM tumor growth in vivo. A) Representative images and B) quantification of relative luciferase on days 7, 14, and 21 post-transplantation; bioluminescence is measured in p-1s-1cm²-1sr. GSC628: shNT (n = 5), shMSN-1 (n = 5), shMSN-2 (n = 5). Data are shown as mean ± SEM. **p* < 0.05, ****p* < 0.001; one-way ANOVA with Tukey’s method for multiple comparisons. C) Kaplan-Meier survival curves of mice bearing GSC628-derived xenografts expressing shNT or shMSN. ****p* < 0.001, log-rank test. GSC628: shNT (n = 10), shMSN-1 (n = 10), shMSN-2 (n = 10). n = 6 (GSC924) or n = 6 (GSC628) biological independent samples.


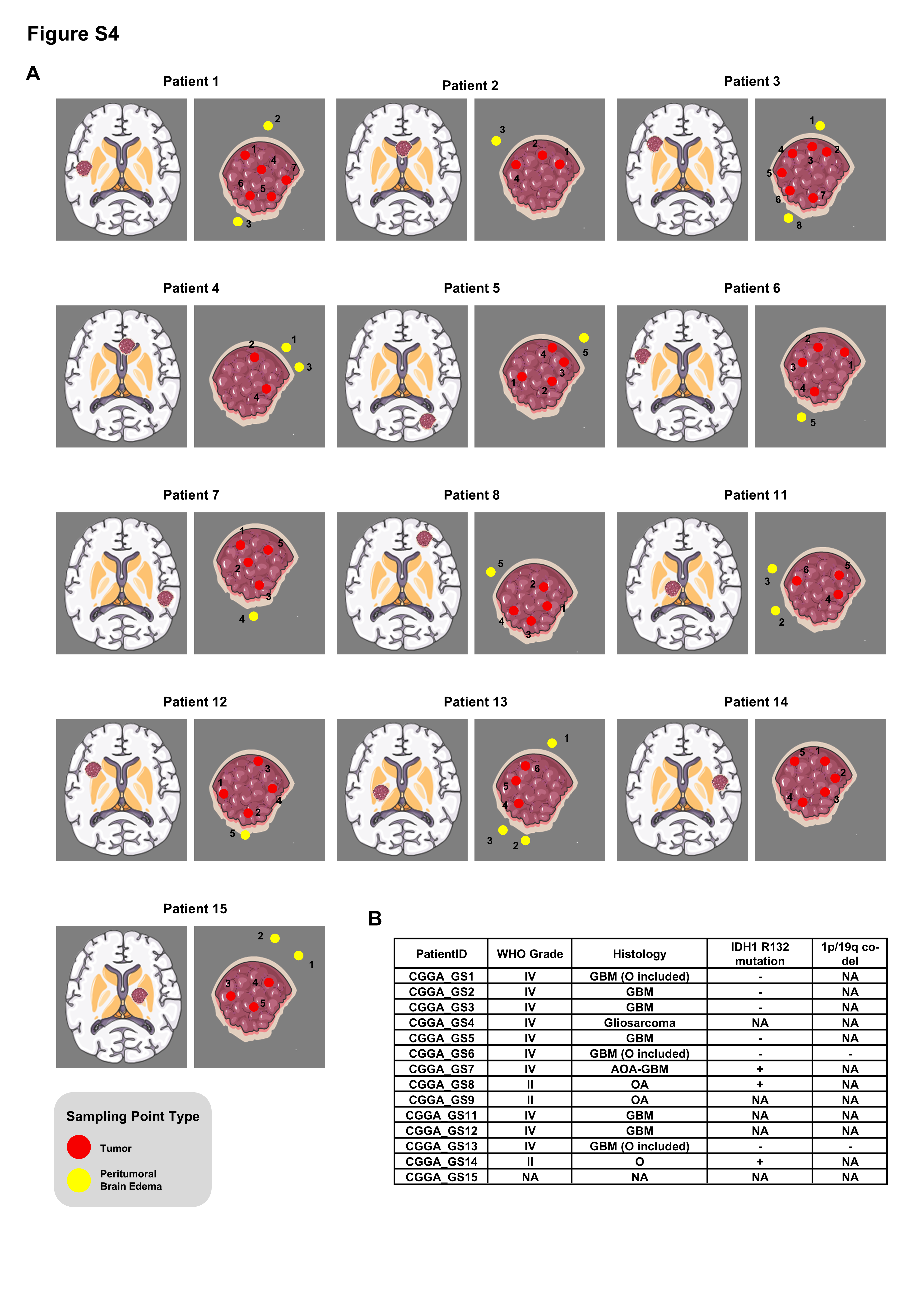


**Figure S4.** Sampling region information for the 14 patients. A) Glioma sampling pattern diagram of in each patient based on MRI. Red and yellow dots indicate tumoral and peritumoral sampling points, respectively. Note: patient 9 had 3 surgery points sampled, but the MRI results were lost. The pattern diagram was created and downloaded using PowerPoint (Microsoft Office 2013). B) Pathology information of the 14 patients collected in this study.

**
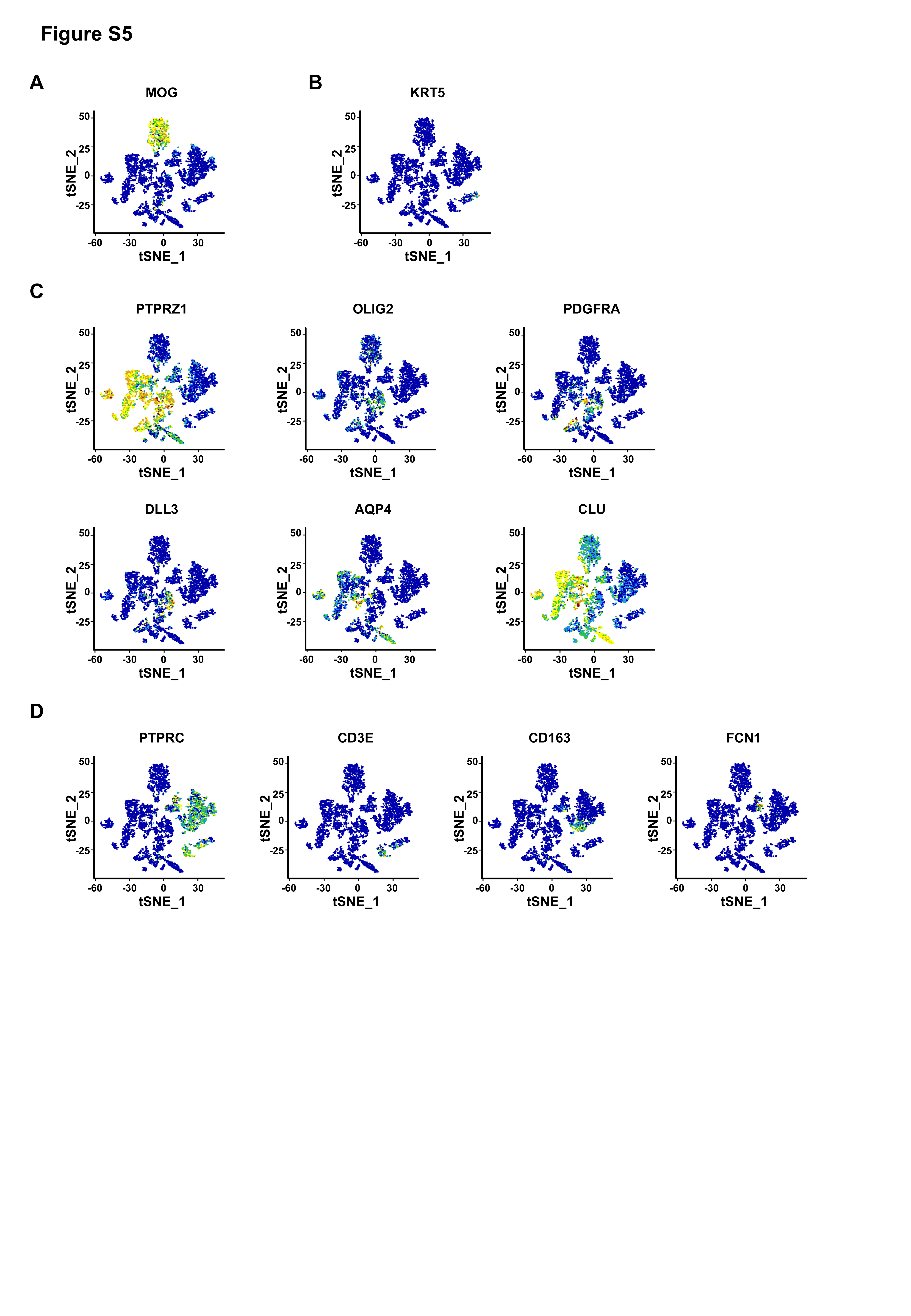
**

**Figure S5.** Patterns of marker gene expression. The distribution of marker genes for A) non-tumor 2 (normal oligodendrocyte), B) lung cancer, C) glioma and D) non-tumor1 (immune cells).


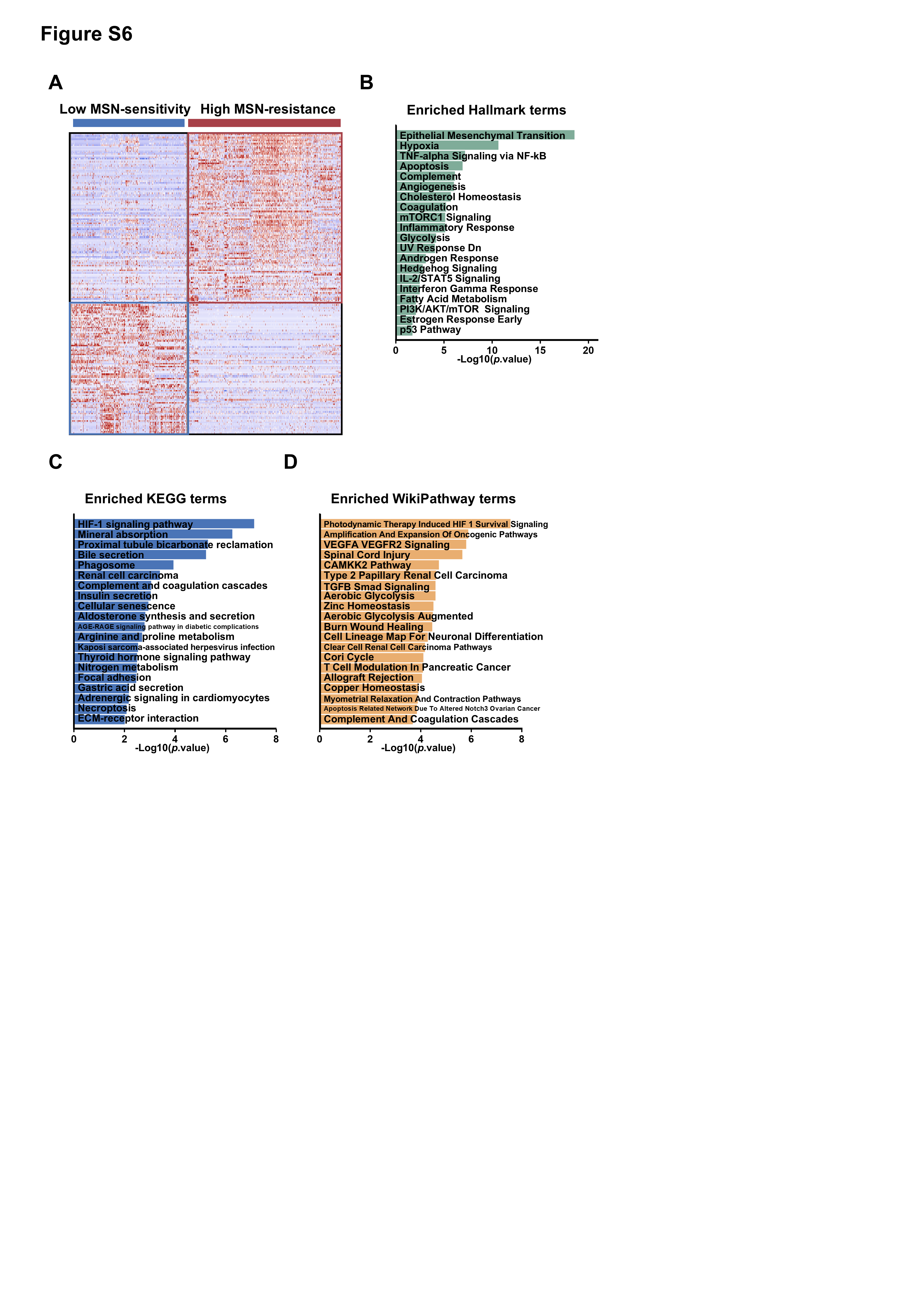


**Figure S6.** The biological characteristics of the high MSN-resistant GBM cluster. A) The heatmap shows the differential gene expression representing the biological characteristics of high MSN-resistant cells and low MSN-sensitive cells. Pathway enrichment analysis of B) Hallmark, C) KEGG, and D) WikiPathway terms.


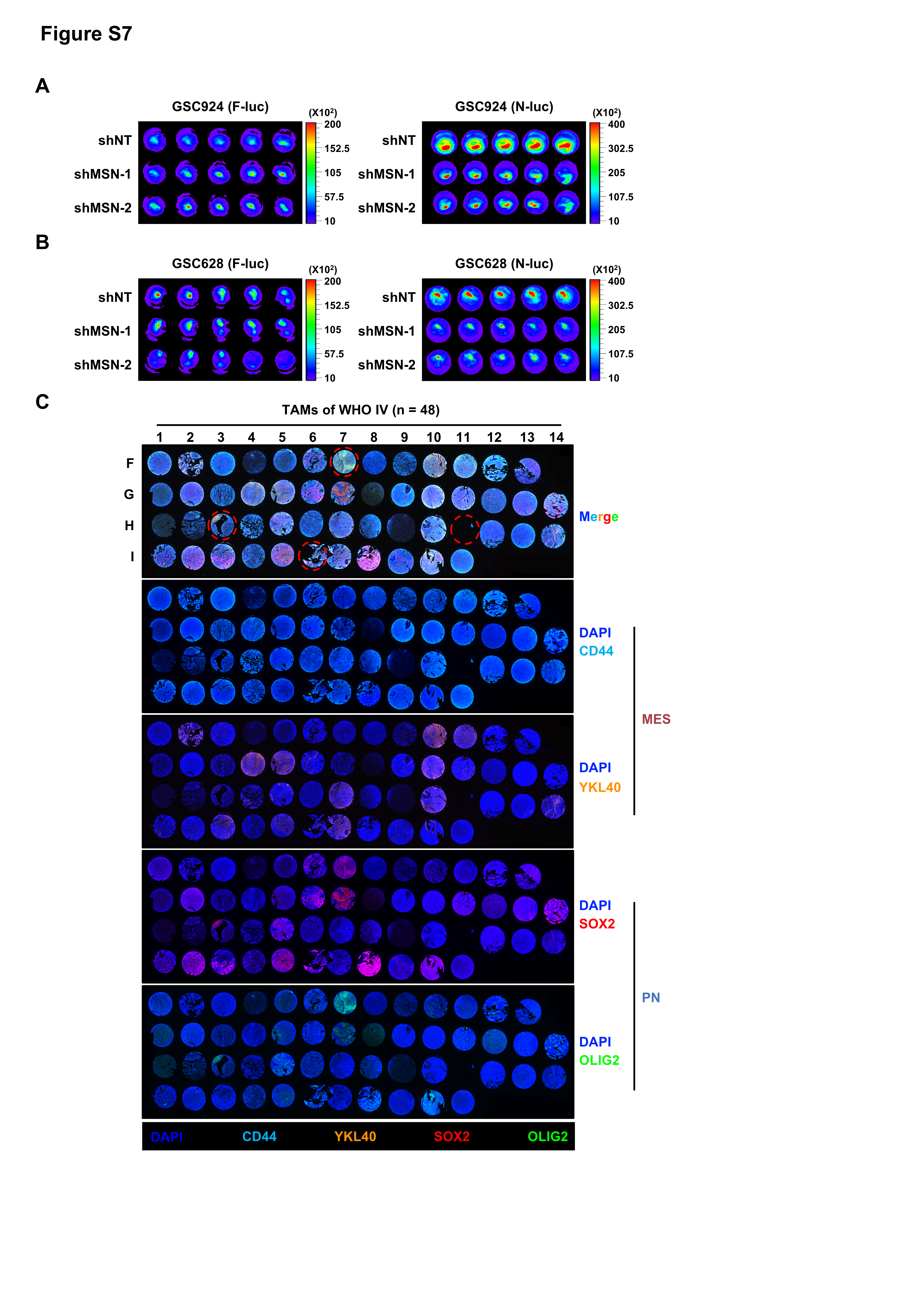


**Figure S7.** MSN is highly expressed in the MES subtype of GBM. CD44 reporter system dual luciferase fluorescence images in (A) GSC924 and (B) GSC628 transduced with shNT or shMSN. C) Multiplex immunofluorescence of MES and PN markers in GBM TAMs. Red circles represent low-quality spots, which are excluded from further analysis.


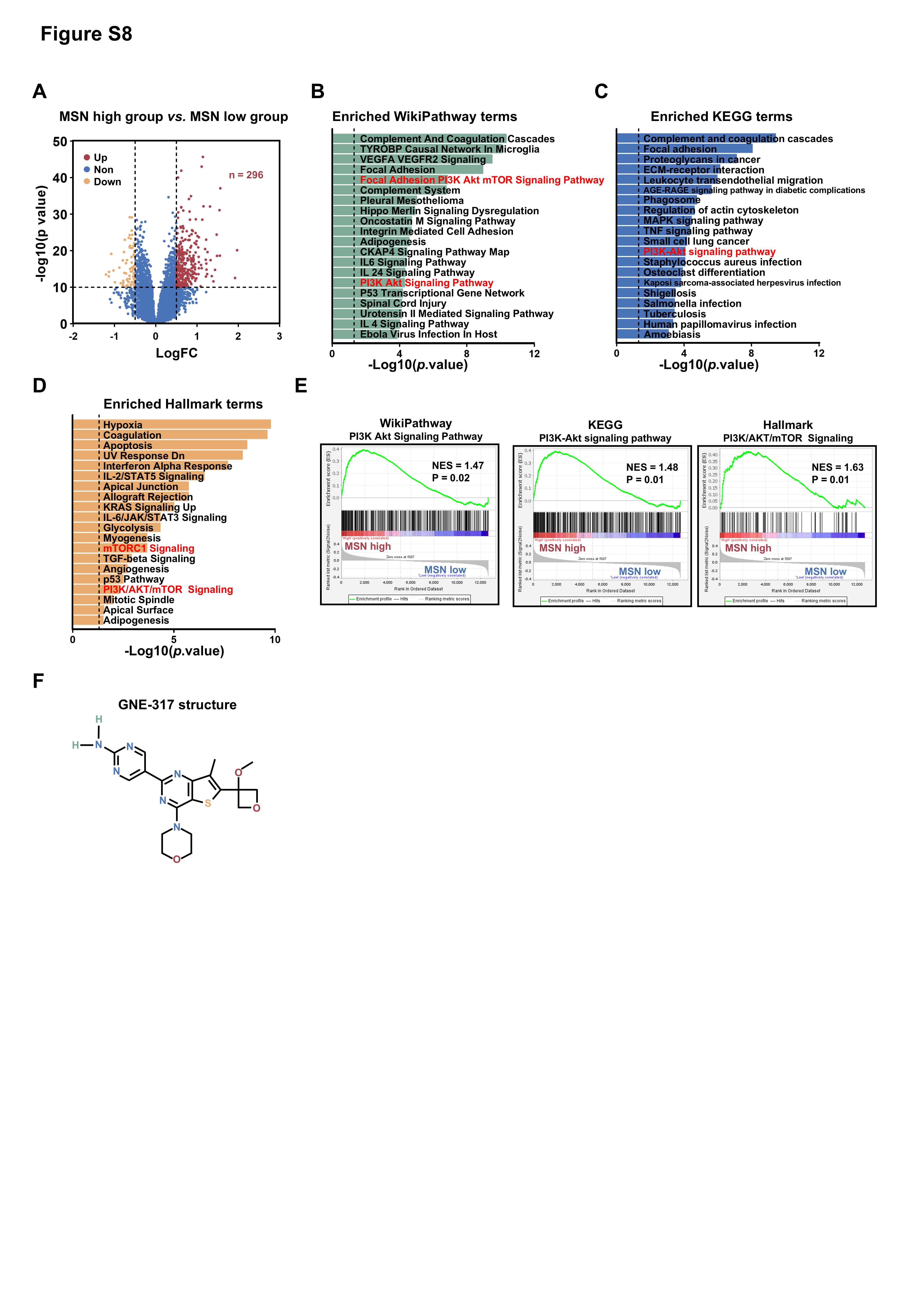


**Figure S8.** MSN may regulate the activation of the PI3K/mTOR signaling pathway in TCGA GBM database. A) The volcano plot shows MSN-regulated differential genes in the TCGA GBM database. Red dots represent MSN upregulated genes, while yellow dots represent MSN downregulated genes. Pathway enrichment analysis of B) WikiPathway, C) KEGG, and D) Hallmark terms. E) GSEA analysis of high-MSN and low-MSN groups in WikiPathway, KEGG, and Hallmark terms. F) The molecular structure of the small-molecule drug GNE-317.


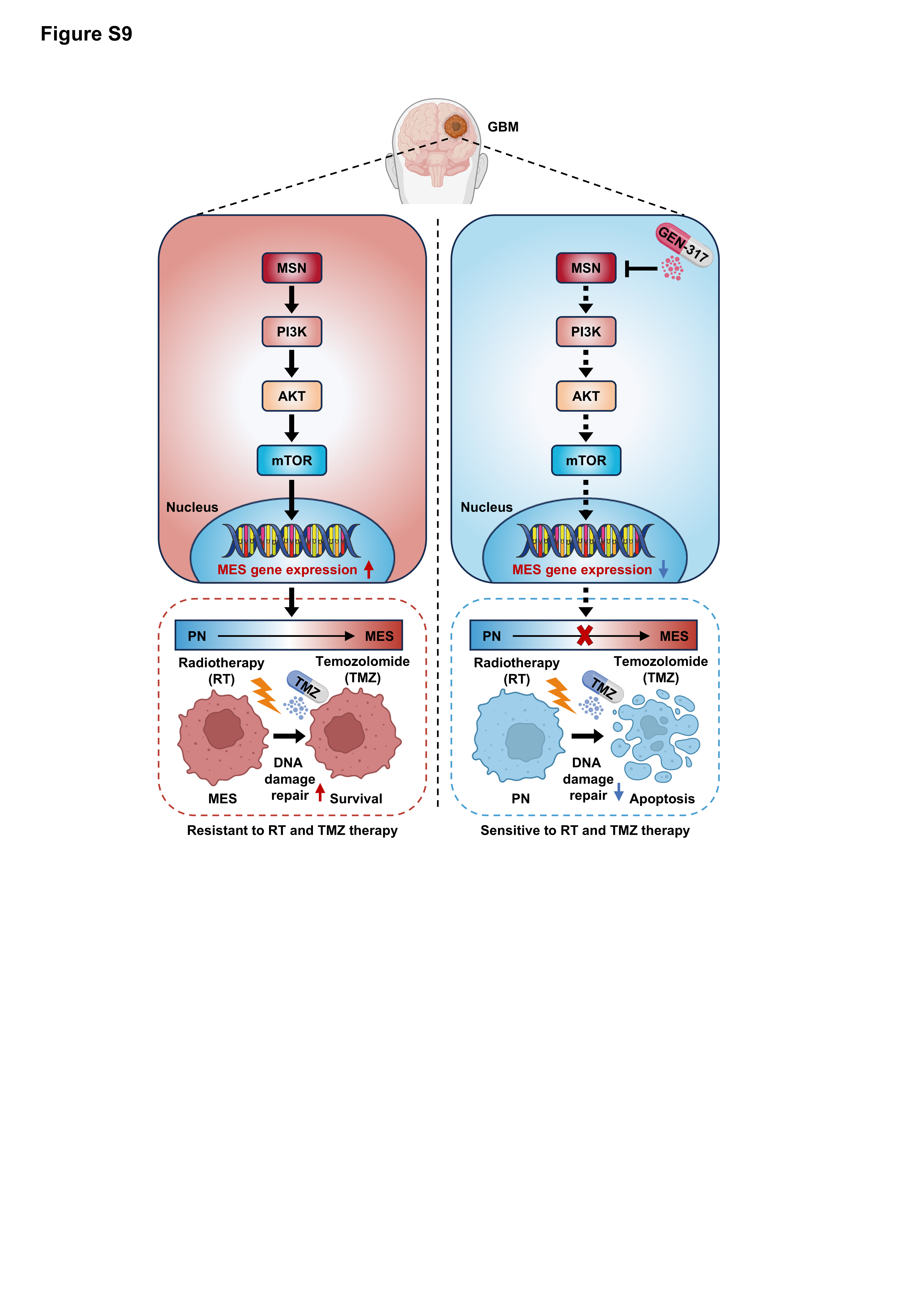


**Figure S9.** The mechanistic scheme of the mechanism of action of MSN and GNE-317. The schematic figure was produced and downloaded by PowerPoint (Microsoft Office 2013).
